# A comparison of optophysiological biomarkers of photoreceptor stress and phototoxicity in BALB/cJ, B6 (Cg)-Tyrc-2J/J, and C57Bl/6J mouse strains

**DOI:** 10.3389/fopht.2023.1128311

**Published:** 2023-03-29

**Authors:** Brent A. Bell, Charles Kaul, Joshua L. Dunaief, Joe G. Hollyfield, Vera L. Bonilha

**Affiliations:** 1 Scheie Eye Institute and Department of Ophthalmology, University of Pennsylvania, Philadelphia, PA, United States,; 2 Cole Eye Institute/Ophthalmic Research, Cleveland Clinic, Cleveland, OH, United States,; 3 Cleveland Clinic Lerner College of Medicine, Case Western Reserve University, Cleveland, OH, United States

**Keywords:** mice, retina, photoreceptors, imaging, photooxidation, phototoxicity, degeneration

## Abstract

Ophthalmic imaging instruments, including the confocal scanning laser ophthalmoscope and spectral-domain optical coherence tomography system, originally intended for revealing ocular microstructures in the human eye, have been deployed by vision researchers to evaluate the eyes of numerous small and large animal species for more than two decades. In this study, we have used these two instruments to obtain imaging data sequentially from the retinas of three prominent, widely used experimental mouse models to document changes induced by two contrasting vivarium lighting conditions. Mice studied include albino BALB/cJ and B6(Cg)-Tyrc-2J/J and pigmented C57Bl/6J. Mice were reared under dim light conditions until ~8 weeks of age where they underwent baseline imaging. Following, mice were returned to the dim vivarium or relocated to the top rack cage position in a standard vivarium. Mice were then followed for several months by ocular imaging to catalog the retinal dynamics as a function of long-term dim vs. elevated, standard vivarium lighting exposure levels. Upon exposure to elevated light levels, B6(Cg)-Tyrc-2J/J underwent similar changes as BALB/cJ in regard to photoreceptor outer segment shortening, photoreceptor layer proximal aspect hyperreflective changes, and the development of retinal infoldings and autofluorescent sub-retinal inflammatory monocyte infiltrate. Noteworthy, however, is that infoldings and infiltrate occurred at a slower rate of progression in B6(Cg)-Tyrc-2J/J vs. BALB/cJ. The photoreceptor outer nuclear layer thickness of BALB/cJ degenerated steadily following elevated light onset. In contrast, B6(Cg)-Tyrc-2J/J degeneration was unremarkable for many weeks before experiencing a noticeable change in the rate of degeneration that was concomitant with a plateau and decreasing trend in number of retinal infoldings and monocyte infiltrate. Pathological changes in C57Bl/6J mice were unremarkable for all imaging biomarkers assessed with exception to autofluorescent sub-retinal inflammatory monocyte infiltrate, which showed significant accumulation in dim vs. elevated light exposed mice following ~1 year of observation. These data were evaluated using Spearman’s correlation and Predictive Power Score matrices to determine the best imaging optophysiological biomarkers for indicating vivarium light stress and light-induced photoreceptor degeneration. This study suggests that changes in proximal aspect hyperreflectivity, outer segment shortening, retinal infoldings and autofluorescent sub-retinal inflammatory monocyte infiltrate are excellent indicators of light stress and light-induced degeneration in albino B6(Cg)-Tyrc-2J/J and BALB/cJ mouse strains.

## 1 Introduction

The mouse (mus musculus) is by far the most popular animal species utilized in biomedical research (1–3). The vast majority of mice used are inbred C57BL/6 and BALB/c strains, which account for ~78% of the total mice used according to a Pubmed search conducted by Festing (4). Biomedical suppliers, academic laboratories and pharmaceutical companies worldwide produce and utilize an astounding number of mice each year to study the cause and effect of human disease. Breeding, rearing, and aging these small animal models of human disease represent a significant cost burden in terms of resources and time for the study of age-related diseases. Hence, being able to identify when a particular model displays the earliest signs of degeneration is of the utmost importance to those searching for interventions that can potentially cure debilitating human diseases. Moreover, it can expedite experimental progress, especially when phenotypes can be subsequently correlated to other key physiological or genetic factors and pharmaceutical or experimental interventions.

Non-invasive ocular imaging instrumentation, namely the confocal scanning laser ophthalmoscope (cSLO) and spectral-domain optical coherence tomography (SD-OCT) have progressed immensely over recent years and are now considered invaluable tools for routine screening of a plethora of small and large experimental animal models. Over the past three decades, these instruments have been indispensable in revealing retinal anatomy in real-time in both human and animal subjects for clinicians and basic scientists alike.

We previously employed these well-established ocular imaging modalities to catalog the dynamic changes in retinal pathology of albino BALB/cJ mice due to vivarium light stress (5). In that study, we found it important to house BALB/cJ mice under dim light conditions and that prominent changes occur rapidly to the retina following relocation and an abrupt change in lighting conditions. The retinas of relocated mice underwent substantial changes in pathology visible by imaging that included the development of photoreceptor layer infoldings and sub-retinal accumulation of autofluorescent inflammatory monocytes. Most importantly, we found that these two imaging phenotypes preceded outer nuclear layer thinning, a hallmark indicator of retinal or photoreceptor degeneration, by several weeks. This previous study: 1) shed light on the delicate nature of working with light susceptible albino BALB/cJ mice, 2) stressed the importance of providing the right vivarium lighting conditions for rearing these animals to minimize light-induced damage, and 3) provided key indicators for detecting when members of this strain are actively experiencing vivarium light-stress.

This previous study also identified one qualitative change observed *via* SD-OCT imaging involving the apical or “proximal” aspect of the photoreceptor outer segments in the BALB/cJ mice undergoing chronic light stress. Other studies detected similar observations from other albino rodents such as B6(Cg)-Tyrc-2J/J mice and Sprague-Dawley rats being housed in our vivarium. Intrigued by these observations we felt it prudent to investigate this phenomenon and determine its viability as a potential imaging phenotype biomarker for vivarium light stress compared to other known imaging indicators of light stress and retinal degeneration including retinal infoldings, autofluorescent sub-retinal monocytes, photoreceptor outer nuclear and outer segment thinning. Similarly, our team has previously reported instances of altered proximal photoreceptor outer segments observed by SD-OCT imaging in the DJ-1 and Nyx-NOB mice due presumably to the influence of oxidative stress and streptozotocin-induced diabetes mellitus (6–8). Others have shown OCT images with similar phenotypes from multiple species including rodents (9–13), canines (14), and humans (15–17); although these changes have been predominantly shown in pigmented as opposed to albino subjects. The changes detected in the “proximal” photoreceptor outer segments are somewhat similar in appearance to Stage 1 changes reported in humans before the development of reticular pseudodrusen (18), thus suggesting a clinical relevance to our previous and current observations made in multiple mouse strains.

The efforts presented here expand on our previous works and analysis (5–8) to include imaging data from three, two albino and one pigmented, commonly employed mouse strains to further validate this novel OCT imaging biomarker and compare its performance to other hallmark indicators of light stress and aging, including the visualization of retinal infoldings, autofluorescent foci (i.e. sub-retinal accumulation of inflammatory monocytes) and photoreceptor degeneration observed by cSLO and SD-OCT imaging, respectively. A secondary objective of this study was to thoroughly characterize the retinal dynamics of these three popular mouse strains over extended periods using two different vivarium lighting conditions. A third mission of this work was to identify which of the imaging biomarkers was best for signaling susceptibility to light stress and determine if any phenotypes are indicative of impending risk for photoreceptor degeneration.

## 2 Materials and methods

### 2.1 Animal use approval and protections

All procedures were conducted under approved animal use protocols by the Cleveland Clinic Lerner College of Medicine Institutional Animal Care and Use Committee and in accordance with the ARVO Statement for the Use of Animals in Ophthalmic and Vision Research.

### 2.2 Mouse strains and experimental objective overview

Breeding pairs of wild type mouse strains, congenic B6(Cg)-Tyrc-2J/J (“B6-albino”; Stock# 000058) and C57BL/6J (“B6”; Stock# 000664), were acquired from The Jackson Laboratory (Bar Harbor, ME). According to JAX Labs, B6-albino mice carry a spontaneous mutation in the tyrosinase gene on a C57BL/6 congenic genetic background and are not considered an inbred strain. Both B6-albino and B6 have the amino acid Methionine at Codon 450 which encodes for the important visual cycle transduction enzyme Retinal Pigmented Epithelium 65 (“RPE65-Met450”). This genetic disposition has been purported to render B6 and B6-albino strains as being protected and partially protected, respectively, from light stress and retinal phototoxicity (19–21). Both B6 strains were acquired to conduct a prospective study in continuation with experiments previously reported pertaining to the retinal dynamics of inbred BALB/cJ mice (“BALB/c”; Stock# 000651) aged under typical or atypical vivarium lighting conditions (5). BALB/c have the RPE65-Leucine to Methionine substitution at Codon 450 (RPE65-Leu450), which is reported to render this strain highly susceptible to light stress and retinal phototoxicity (19–22). To compare results from all three mouse strains, BALB/c data reported in Bell et al., (5) underwent a retrospective analysis identical to those developed for the prospective experiments conducted in B6 and B6-albino mice. The results obtained from these three common mouse strains have been analyzed similarly and are displayed together for comparison.

### 2.3 Confocal scanning laser ophthalmoscope (cSLO)

A model HRA2 cSLO (Heidelberg Engineering, Inc., Franklin, MA) was used to collect retinal fundus photos using Infrared reflectance (IR) and Blue autofluorescence (BAF). A 55° wide-field lens was used to collect images with the optic disk centrally located. Techniques for optimal collection of subretinal pathology including retinal infoldings and autofluorescent foci (e.g. sub-retinal inflammatory cells) have been previously reported in Bell et al., (5).

### 2.4 Spectral-domain optical coherence tomography (SD-OCT)

Structural *in vivo* imaging of the posterior pole was performed using a Bioptigen SD-OCT system (Model SDOIS, Leica Microsystems, Buffalo Grove, IL). Imaging system theoretical (in air) lateral and axial resolution is a few (~2.5) to several (~7) microns, respectively. A Bioptigen mouse bore objective lens with a 50° field of view (FOV) was used for posterior pole imaging with an estimated lateral FOV of ~1.5 mm. In BALB/c mice, B-scans (1000 A-scans/B-scan, 1B-scan, 15 frames) were collected from both eyes through the horizontal meridian with the optic disk centrally positioned in the *en face* view. For B6 and B6-albino mice, orthogonal B-scans of the horizontal and vertical meridians of posterior pole with optic disk centered were collected using radial volume scan with parameters of 1000 A-scans/B-scan by 2 B-scans by 15 frames.

### 2.5 Experimental procedures

Experimental procedures employed are detailed in Bell et al., (5). Briefly, mice underwent a short quarantine period upon delivery from the vendor and were subsequently moved to a low-illumination (~1 lx; 14/10 hrs. light/dark) vivarium. Resulting progeny produced in this vivarium were weaned at ~3 weeks of age and separated by gender into individual cages with up to four mice per cage. At 9 weeks of age, mice were removed from the low-illuminance vivarium and transported to the ocular imaging facility for baseline imaging with cSLO and SD-OCT. Mice were then divided into two (B6 and B6-albino) or three (BALB/c) cohorts for aging and long-term, synchronous or asynchronous, follow-up imaging assessments while housed under either low or standard illuminance cyclic (14/10 hrs. day/night) lighting conditions. Table 1 shows relevant details related to vivarium room type, cage rack location, intracage illumination intensity, mouse strains investigated and follow-up imaging timepoints.

As previously reported in Bell et al., (5), BALB/c mice were randomly divided into three groups (Grps A, B & C) and placed into either the low illuminance (Grp A) or standard illumination rooms (Grps B & C). In the standard vivarium room, mice were permanently housed on either the bottom (Grp B) or top (Grp C) shelves of ventilated cage racks. Intracage illumination levels (Mean ± SD) for Grps A, B, and C were 1.2 ± 1.7, 7 ± 7, and 151 ± 79 lx, respectively.

Mice from Grps B & C were reimaged multiple times over the course of 48 wks while multiple cohorts of Grp A mice were randomly imaged only once up to 96 wks post-baseline. Only data for Grps B & C (n=37; 17 male/20 female) were analyzed retrospectively to compare with the prospective study results involving B6 and B6-albino mice. The scope of the retrospective analyses was limited to Grp B vs. C as no appreciable differences in photoreceptor degeneration were documented between Grp A vs. B in Bell et al., (5).

As shown in Table 1, the three vivarium possibilities used will herein be referred to as dim light exposure (DLE) or bright light exposure (BLE) treatments instead of Groups A, B, & C. Additional information in parentheses describes the vivarium and cage rack locations as low illuminance random (LIR), standard illuminance bottom (SIB), & standard illuminance top (SIT) and the mean intracage illuminance levels in lux (1, 7 or 150 lx).

Following baseline, B6 (n= 27; 14 male/13 female) and B6-albino (n= 78; 40 male/38 female) mice were either returned to the low-illuminance vivarium for chronic DLE (LIR-1 lx) exposure or placed on a top row of the ventilated cage rack in the standard illumination vivarium for chronic BLE (SIT-150 lx) exposure treatment. Over the following year, B6 and B6-albino mice were removed from their respective vivaria for transport to the *in vivo* imaging lab for follow up, synchronous or asynchronous, retinal imaging up to 70 months post-baseline, respectively. Imaging lab illuminance was ~300–350 lx at desk level and no precautions were taken to shield animals from ambient lighting. Imaging experiments were conducted between the hours of 9 AM to 4 PM and all mice returned to their respective vivarium that same day.

### 2.6 Short-term testing for reversibility of light-stress induced photoreceptor layer changes

An experiment was devised to determine whether the reflective changes observed in the photoreceptor layer of B6-albino mice were permanent or reversible following removal of the bright light stimulus. To accomplish this, mice underwent a Dim-Bright-Dim light exposure challenge, which we will refer to as DLE-BLE-DLE. Individual cohorts of B6 and B6-albino mice were housed in dim cyclic lighting conditions (LIR-1 lx) from birth to 9 weeks of age. At 9 weeks of age, mice were removed from the dim vivarium, imaged by cSLO and SD-OCT, and then returned to the same vivarium location. This process was repeated for one additional week and upon completion of the second imaging session, mice were immediately transferred to another vivarium (SIT-150 lx) for BLE. A week later, the cohorts were again removed, imaged, and returned (SIT-150 lx) for another week of BLE. Upon completion of another imaging session following another week of BLE, cohorts were then transferred back to the dim vivarium (LIR-1 lx). Following this final transfer, cohorts were imaged an additional two times over the following two weeks under DLE conditions (LIR-1 lx).

### 2.7 Data processing and analysis

#### 2.7.1 Analysis of retinal infoldings, autofluorescent foci and retinal degeneration

Procedures for analyzing cSLO and SD-OCT images for retinal infoldings (RIF; Analysis #1), autofluorescent foci (AFF; Analysis #2) and photoreceptor outer nuclear layer (ONL; Analysis #3) degeneration has been previously described (5) and are detailed in Table 2. Briefly, cSLO images from both eyes of each mouse were exported as TIFF files from the Heidelberg HRA2 imaging platform and displayed in ImageJ (23). Infrared reflectance (IR-cSLO) and blue peak autofluorescence (BAF-cSLO) images were assessed for RIF and AFF using the manual counting tool in ImageJ.

SD-OCT B-scans of retinal meridians from both eyes were exported as.AVI files and opened in ImageJ. Fifteen frames from each AVI file was co-registered and averaged using StackReg/TurboReg plug-in (24). Outer nuclear layer thickness was measured half-way from the optic nerve to the image margin. For B6 and B6-albino mice, ONL measures were obtained from all four retinal regions (temporal, nasal, inferior, & superior) whereas in BALB/c mice, only data from temporal and nasal regions were obtained.

#### 2.7.2 Analysis of SD-OCT B-scans using longitudinal reflectance profiles

Longitudinal reflectance profiles (LRP) were obtained as previously described (6–8) to perform Analyses #4–7. Briefly, LRP regions of interest (ROI) 40 pixels wide were collected on either side of the optic nerve midway from the center to the edge of B-scan window margin. ROIs were obtained from both sides of the optic nerve from the temporal and nasal regions of BALB/c mice, and from all four quadrants (temporal, nasal, inferior, and superior regions) of B6 and B6-albino mice. ROIs were averaged into a single linear profile that showed average back-reflected signal amplitude as a function of axial imaging depth. Resulting LRPs were assessed for changes in photoreceptor layer signal reflectivity (Analyses #4–6) and morphology (Analysis #7) as detailed in Table 2.

Analysis #4, illustrated in Supplemental Figure S1, assessed the photoreceptor outer segments for changes in the reflective signal slope (OSS∠) as previously described (6). Analysis #5, illustrated in Supplemental Figure S2, assessed changes in the back-reflected signal intensities (ΔOS) originating from the photoreceptor outer segments minima (OS_min_) relative to the photoreceptor inner/outer segments transition maxima (IS/OS_max_). Analysis #6, illustrated in Supplemental Figure S3, assessed changes in the back-reflected signal intensities (ΔIS) originating from the photoreceptor inner segments minima (IS_min_) relative to the photoreceptor inner/outer segments transition maxima (IS/OS_max_).

Illustrated in Supplemental Figure S4, Analysis #7 obtained additional morphological information beyond those collected for Analysis #3 for the changes in ONL thickness. Analysis #7 was accomplished using three measurement passes through the LRP data. LRP contours, with their undulating minima and maxima, permitted clear visualization of various lamina within the outer retina and choroid. Axial distances between laminar features such as the outer limiting membrane (OLM), photoreceptor inner and outer segments transition (IS/OS), photoceptor distal outer segments (OS), the retinal pigment epithelium (RPE) and Bruch’s membrane (BM) together referred to as the retinal pigment epithelium-Bruch’s membrane complex (RPEBM), were measured and manually subtracted to yield: 1) Analysis #7 – the photoreceptor layer (PL) thickness, 2) Analyses #7a & 7b - the subdivision of PL to provide separate thicknesses for the photoreceptor inner segments (IS; 7a) and photoreceptor outer segments plus retinal pigment epithelium (OS+RPE; 7b), and 3) Analysis #7b.1 & 7b.2 – which further subdivided the OS+RPE into the OS (7b.1) and RPE (7b.2).

Each eye was considered a single sample. Measures from the various quadrants of each retina (BALB/c: Temporal & Nasal; B6 & B6-albino: Temporal, Nasal, Inferior, & Superior) were averaged together to provide a single mean ± standard deviation (SD) per eye.

### 2.8 Graphical presentation of data and biostatistics

Graphics and statistical analysis were accomplished using GraphPad Prism 9 (Graphpad Software, La Jolla, CA). Unless otherwise noted, datapoints are shown in figures as mean± SD. Dynamic changes in RI and AFF count, OSS∠, ΔOS, ΔIS, and ONL, PL, IS, OS, OS+RPE, and RPE thickness were displayed using a polynomial curve (1st, 2nd or 3rd order best-fit showing the mean ±95% confidence interval and R-squared value).

For 9 wk of age baseline comparisons between mouse strains under DLE conditions an Ordinary One-way ANOVA with Holm-Sidak’s multiple comparisons test was performed. For evaluation of one-week post-baseline transfer of mice from DLE to BLE a Two-way ANOVA with Sidak’s multiple comparisons test was performed. A multiple t-test corrected for multiple comparisons using the Holm-Sidak method was used to determine statistical significance for synchronous measures obtained between BLE vs. DLE groups. P values or adjusted p values are numerically written in the manuscript and displayed in the graphs with asterisks using the following convention: ns = not significant; *p < 0.05; **p < 0.01; ***p < 0.001; ****p < 0.0001.

Nonparametric Spearmen’s ρ correlation test and Predictive Power Score (PPS or PPscore) matrices (25) was performed individually on each mouse strain data set using GraphPad Prism and Python v3.8.2 programming language, respectively. Spearmen’s correlation strengths followed typical nomenclature for this test and will be referred to as follows: very strong (0.8–1.0), strong (0.6–0.79), moderate (0.4–0.59), weak (0.20–.39), and very weak (0.0–0.19). The predictive power score computes whether a “feature” (i.e. variable) can predict another feature referred to as the “target”. The score is an asymmetric, data-type-agnostic score for predictive relationships between two or more columns of data, ranging from 0 to 1. A score of “0” implies that the feature column x cannot predict the target column y better than a naive baseline model. A score of “1” implies that the feature column x can perfectly predict the target column y given the model. Calculations are based upon the decision tree learning algorithm for classification. Unlike a typical correlation, this method will show the extent of asymmetrical patterns within the data. PPS does not have any defined nomenclature to describe the 0.0–1.0 scale and thus we have elected to use the same scale that is employed for the Spearman’s correlation to describe the PPS results.

## 3 Results

Prospective experiments were successfully performed on cohorts of B6 and B6-albino mice similar to those previously reported in BALB/c (5). cSLO and SD-OCT consistently provided informative images in all three strains of mice related to vivarium illumination level and exposure time.

### 3.1 Analyses 1–3: Retinal infoldings, autofluorescent foci and photoreceptor outer nuclear layer degeneration between BALB/c, B6-albino and B6 mice

**FIGURE 1:**
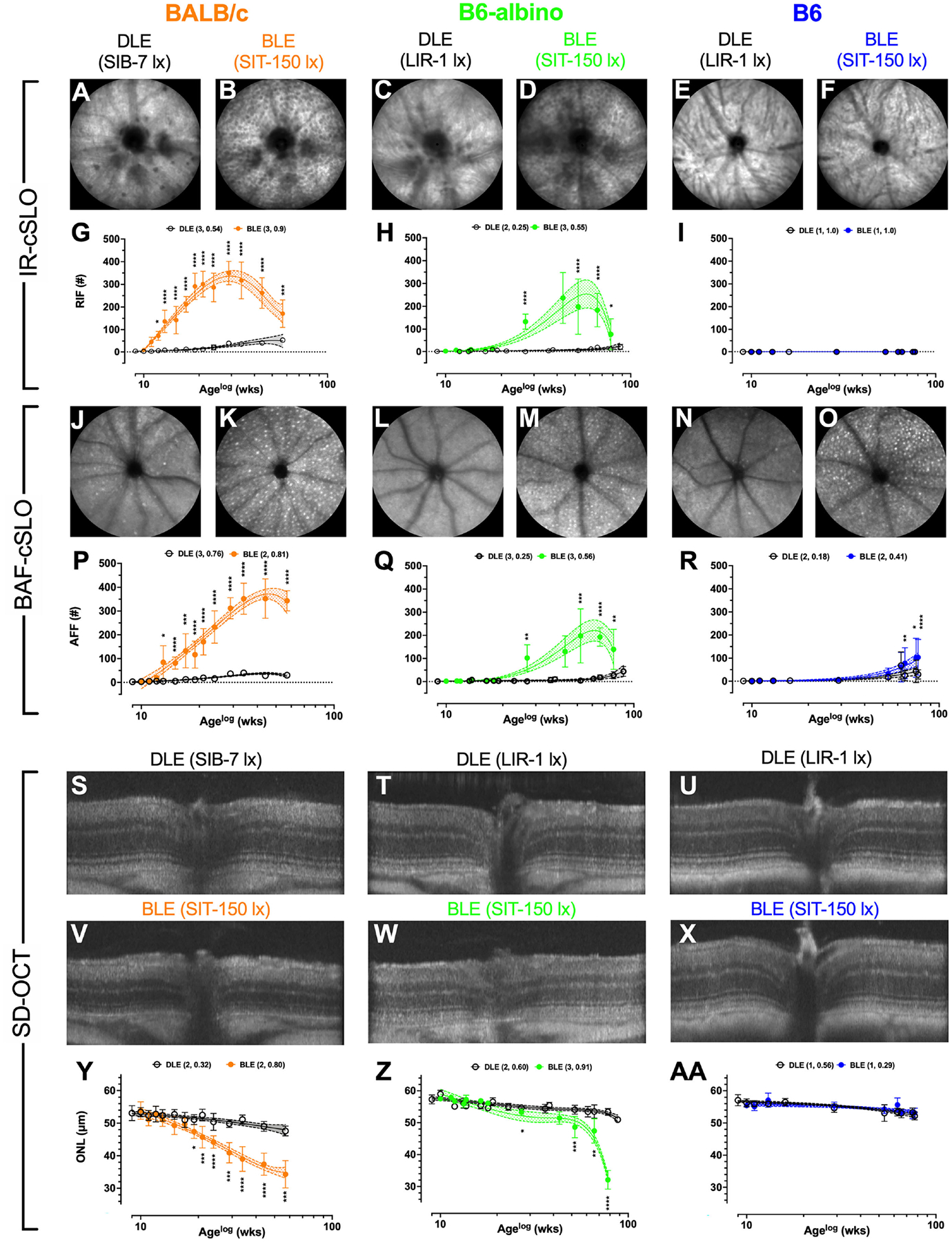
Results for Analyses #’s 1–3 showing temporal changes in Retinal Infoldings **(A**–**F; G**–**I)**, Autofluorescent Foci **(J**–**O; P**–**R)** and Photoreceptor Degeneration **(S**–**X; Y**–**AA)** for BALB/c, B6-albino and B6 mice after chronic exposure to DLE and BLE conditions. BALB/c images are from 24 wks (Age: 33 wks) post exposure onset. B6-albino and B6 mice images are from 57 wks (Age: 66 wks) post exposure onset. Both albino strains developed substantial numbers of RIF which were not observed in pigmented B6 mice. All three strains developed significant AFF that varied in progression rate relative to light exposure duration. Significant photoreceptor degeneration was observed in the two albino strains only, with substantially different decay rate behaviors witnessed between the two albino strains as BALB/c and B6-albino exhibited uniphasic and biphasic decays, respectively. BLE and DLE legends display in parenthesis the best fit polynomial order (i.e. 1, 2, or 3) and R-squared value.

An initial objective of this study was to document the presence or absence of RIF, AFF and photoreceptor degeneration (i.e. ONL thinning) in two additional wildtype mouse strains relative to observations made previously in BALB/c (5). Our previous work demonstrated that BALB/c, housed under bright vs. low-illuminance conditions, develop significant RIF (Figure 1B; p=.004 @ 3 wks) and AFF (Figure 1K; p=.0002 @ 4 wks) prior to significant ONL thinning (Figure 1Y; p=.0038 @ 10 wks) (5). Similar to BALB/c, B6-albino mice also developed RIF, followed thereafter by the development of AFF, and a delayed onset of ONL thinning. Significant changes with each of these indicators was documented in both albino strains, albeit occurring at different rates of progression. In contrast, the only changes observed in the pigmented B6 strain was related to AFF.

Representative IR-cSLO (Figures 1A–F), BAF-cSLO (Figures 1J–O) and SD-OCT (Figures 1S–X) images from each mouse strain are shown demonstrating the retinal dynamics of RIF and AFF count and ONL thickness. For IR-cSLO, differences in RIF count are apparent between Dim and Bright light conditions for both BALB/c (Figures 1A vs. B) and B6-albino (Figures 1C vs.D) strains. RIF was not detected in B6 mice (Figures 1E vs. F). Temporal changes in RIF count are shown in Figures 1G–I for BALB/c, B6-albino, and B6 strains, respectively. Significant differences in RIF count were observed at 3 wks for BLE vs. DLE BALB/c mice (Figure 1G). Figure 1G shows that BALB/c accumulated RIF at a rate of ~16 per week, peaking with ~350 total at 20 weeks (Age 29 wks), and began to decline thereafter at ~7 per week. RIF count in B6-albino mice behaved similarly to BALB/c, with exception to a slower rate of development and progression (Figures 1G vs. F). As shown in Figure 1H, a significant RIF count between BLE vs. DLE groups was not observed until ~18 wks (Age 27 wks) post-BLE onset. Figure 1H also shows that B6-albino accumulated RIF at a rate of ~6 per week, peaking with ~250 total at ~50 weeks (age 59 wks), and then began to decline at ~8 per week. No RIF data was obtained for B6 mice since they were not observed to occur (Figure 1I).

For BAF-cSLO, differences in AFF count was observed between Dim and Bright light conditions for BALB/c (Figures 1J vs. K), B6-albino (Figures 1L vs. M), and B6 (Figures 1N vs. O) strains. Temporal changes in AFF count are shown in Figures 1P–R for the three mouse strains. Significant differences in AFF count were observed at 4 wks for BLE vs. DLE BALB/c mice (Figure 1P). Figure 1P also shows that BALB/c accumulated AFF at a rate of ~11 per week, peaking with ~350 total at 35 weeks (age 45 wks), and did not decrease after 15 more weeks. AFF in B6-albino mice behaved similarly to BALB/c (Figures 1Q vs. P), albeit with a lower total peak count and a slower rate of progression. Similar to RIF count (Figures 1H vs. Q), a significant AFF count between BLE vs. DLE B6-albino groups was reached at ~18 wks (age 27 wks) post-exposure onset. Figure 1Q shows that B6-albino accumulated AFF at a rate of ~5 per week, peaking with ~200 total at ~50 weeks (age 60 wks) post-exposure onset, and then began to decrease at ~4 per week. Surprisingly, a significant difference in AFF count was observed between BLE vs DLE groups for aged B6 mice (Figure 1R). B6 mice housed under BLE conditions accumulated AFF at ~1.4 per week and became significant over the DEL group after 57 wks of BLE exposure. After 70+ weeks of exposure, B6 mice housed under BLE conditions had twice as many AFF as their DLE counterparts.

Representative SD-OCT images of DLE and BLE treatment groups from each mouse strain is shown in Figures 1S–X. Significant photoreceptor degeneration occurred between BLE vs. DLE groups for BALB/c (Figures 1V vs. S) and B6-albino (Figures 1W vs. T) strains, but not the B6 (Figures 1X vs. U) strain. Notable between BALB/c and B6-albino is the degeneration kinetics, with BALB/c decaying consistently with time and B6-albino experiencing a latent, rapid onset of ONL thinning after ~ 40 wks of BLE exposure (Figures 1Y vs. Z). In BALB/c (Figure 1Y), photoreceptor degeneration occurred in the BLE group at a steady rate of ~0.36 μm/week following light exposure onset and became significant relative to the DLE group at ~8 wks (age 17 wks). For comparison, the rate of ONL thinning in BALB/c DLE group was ~0.1 μm/week. In the B6-albino strain (Figure 1Z), the ONL thickness of the BLE group trended thinner than the DLE group between 9–40 wks (age 20–50 wks) following exposure onset. After 40 wks (age ~50 wks), ONL thickness rapidly decreased through the last data collection time point of 70 wks (age ~80 wks) post-exposure onset. The mean rate of ONL thinning from 9–40 wks post-exposure onset for BLE vs. DLE B6-albino groups was ~0.175 vs. ~0.075 μm/week. After 40 wks of exposure, the rate of ONL thinning for the BLE group increased by 5.7x to ~0.567 μm/week while the rate in the DLE group only doubled to ~0.133 μm/week. B6 mice did not exhibit significant ONL thinning between BLE and DLE groups (Figure 1AA). Collectively, the rate of ONL thinning was ~0.062 μm/week for the BLE and DLE groups combined.

**FIGURE 2:**
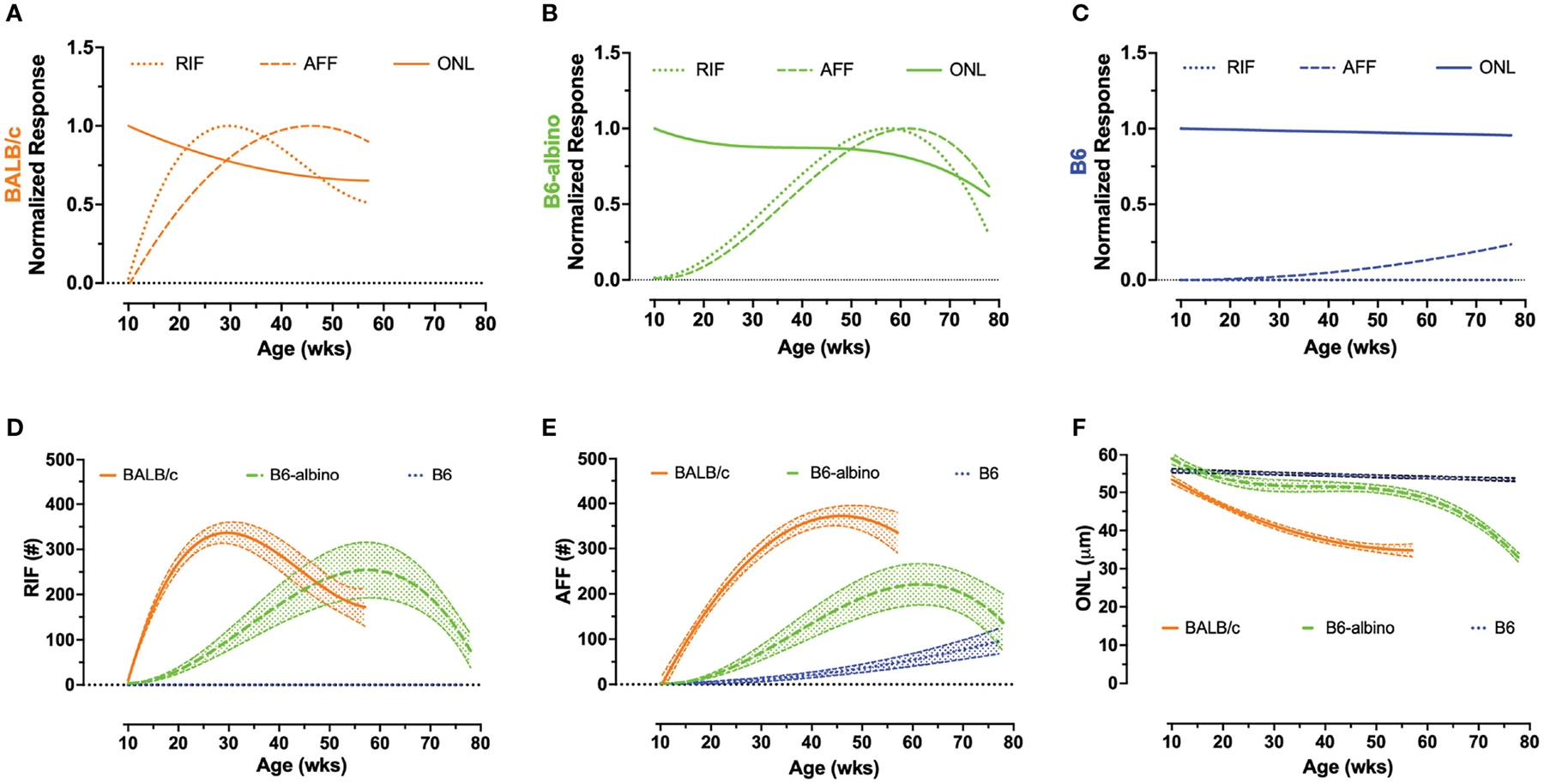
Intra- **(A**–**C)** and inter-strain **(D**–**F)** summary graphs showing the retinal dynamics of RIF, AFF and photoreceptor degeneration (ONL) in BALB/c, B6-albino and B6 mice. I ntra-strain biomarker comparisons of normalized mean dynamic trends for RIF, AFF, and photoreceptor (ONL) degeneration in BLE cohorts of BALB/c **(A)**, B6-albino **(B)** and B6 **(C)** mice. Inter-strain biomarker comparisons of non-normalized RIF **(D)**, AFF **(E)**, and photoreceptor (ONL) degeneration trends (mean count/thickness +/− CI) in BLE mouse cohorts.

The logarithmic (LOG) time scale used for the abscissa in Figures 1G–I, P–R, Y–AA showed the biomarker datapoints and mean trends well-distributed over the course of the study. This display format was good for demonstrating when a biomarker became significant at the earliest moment following BLE onset. Displaying the data in a linear manner would have compressed all datapoints during the early period of this study and made discerning significant changes difficult. Nevertheless, one limitation of the LOG display format is that it makes it difficult to compare the long-term temporal aspects of the data over time, especially when discussing rates of change between strains of mice. Figure 2 was created summarize and compare these three biomarker trends for the BLE exposed cohorts. These additional figures addressed the limitation of displaying the data in LOG format and improve data visualization of retinal dynamics (i.e., rates of change) between biomarkers (e.g., RIF, AFF and ONL) and individual mouse strains (e.g., BALB/c, B6-albino and B6). Figures 2A–C shows the intra-strain comparisons of normalized RIF and AFF count and ONL thickness with a linear abscissa. In these graphs, biomarker magnitude trends have been normalized with respect to maximum and minimum counts (RIF & AFF) or thickness (ONL) observed over the study duration. From these graphs one can better discern the interplay between biomarker dynamics and the rates of change over time. From this we can see in Figure 2A that the ONL trend in BALB/c is steadily decreasing from BLE onset but does appear to be approaching an asymptote at ~70% of full retina thickness. The slowing ONL degeneration rate appears to coincide with peak roll-off and downward inflections of RIF and AFF count. This could indicate that accommodation and adaptation of the retina is occurring with animals being maintained at this consistent BLE illuminance level (~150 lx). In contrast, Figure 2B demonstrates how the rate of RIF and AFF in B6-albino are tracking together in count before ONL thickness begins to change in conjunction with the maximum and roll-off of RIF and AFF count. Figure 2C shows the absence of RIF and the lack of change in ONL thickness in response to an increase in AFF count following long-term exposure of B6 mice to BLE.

Figures 2D–F shows the mean trends +/− 95% C.I. for RIF (Figure 2D) and AFF (Figure 2E) counts and ONL thickness (Figure 2F) overlaid for comparison among the three strains of mice. As can be clearly observed in Figures 2D–E, the rate of development of RIF and AFF is higher for BALB/c than B6-albino, demonstrating that the former strain has an increased propensity for developing these features over the latter when both are housed under the same BLE conditions. Figure 2F compares the ONL degeneration measured between the three strains of mice. In this figure B6 shows no degeneration following BLE onset and over the course of the study whereas BALB/c clearly degenerate immediately and significantly within a few weeks following BLE onset. In-between B6 and BALB/c, a hybridized degeneration trend is observed for B6-albino. According to the non-linear regression curve obtained on B6-albino data, this strain perceivably undergoes some initial thinning between 10 and 20 weeks, followed thereafter by a long period of accommodation and stabilization from 20 to 60 weeks, then at 60 weeks or age a rate of change in degeneration is observed following ~1 year of BLE exposure.

### 3.2 Analysis 4–7: Longitudinal reflectance profiles (LRP) for the analysis of signal amplitude and morphological changes in the photoreceptor layer

**FIGURE 3:**
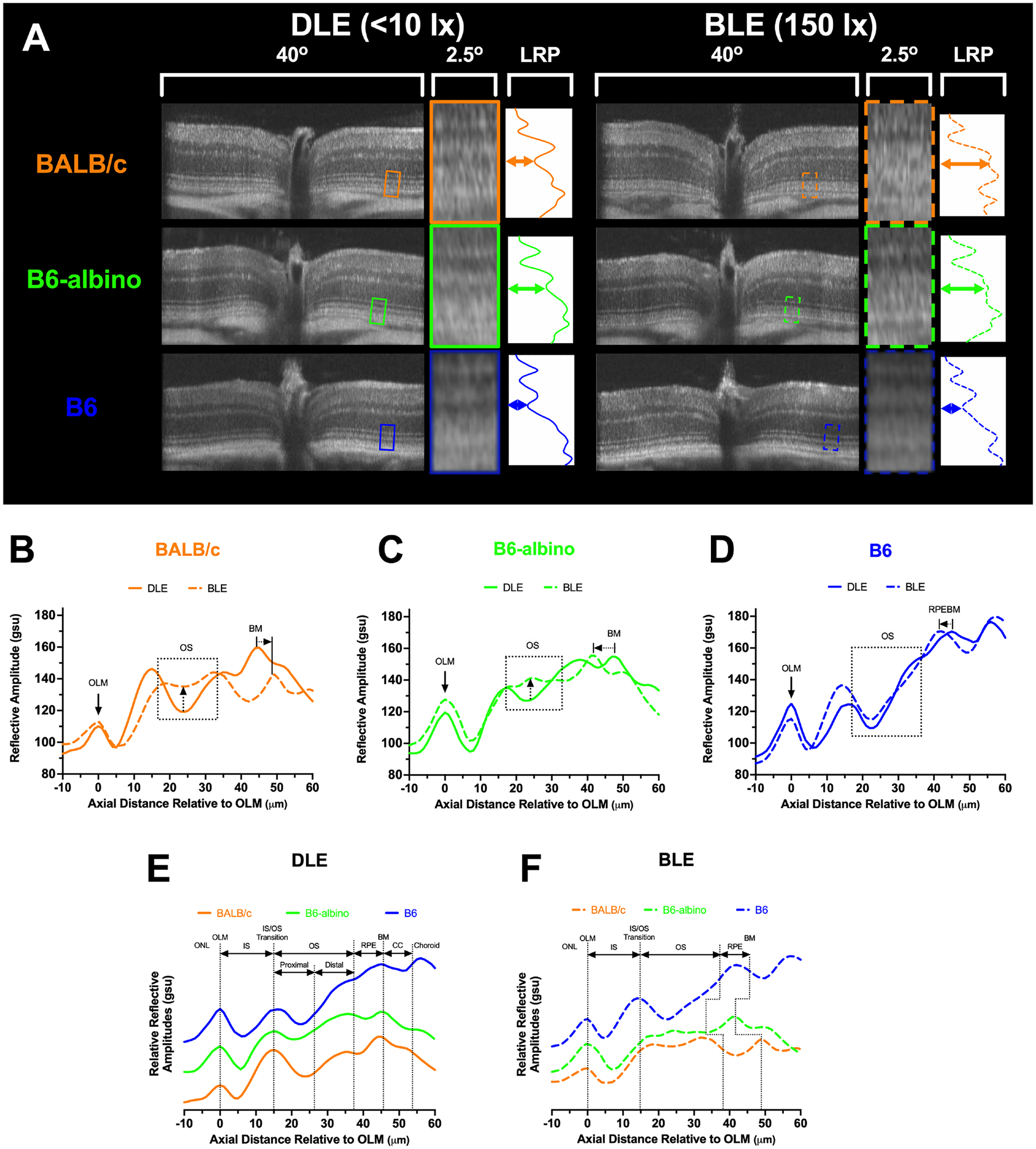
SD-OCT images and Longitudinal Reflectance Profile (LRP) examples from DLE and BLE retinas of BALB/c, B6-albino, and B6 mice. **(A)** Co-registered and averaged 50° FOV (~1.5 mm width) B-scans from the horizontal meridian detailing the outer retina 2.4° region of interest (ROI). The ROI is a 40 pixel-wide window that is averaged to generate the Longitudinal Reflectance Profile (LRP). The LRP displays the intensity vs. axial distance of the photoreceptor layer with a small portion of adjacent ONL and choroid. Location of normal or altered proximal outer segments under DLE or BLE conditions respectively, denoted by colored horizontal double arrows in the LRP examples. **(B**–**D)** LRPs are rotated CCW 90 degrees, aligned to the OLM and qualitatively compared to reveal differences between DLE and BLE conditions for BALB/c **(B)**, B6-albino **(C)** and B6 **(D)** strains. Changes in proximal OS signal reflectivity are accentuated with up-directional arrows for BALB/c and B6-albino mice housed in DLE vs BLE conditions. Changes in photoceptor layer thickness induced by BLE vs DLE can also be observed by looking at the relative locations of the RPEBM or BM for pigmented and albino mice, respectively. LRP comparisons between mouse strains for DLE **(E)** vs. BLE **(F)** conditions showing the proximal and distal author defined designations for the photoreceptor outers segments relative to the widely accepted anatomical locations for a mouse retina SD-OCT B-scan. DLE housed mice show consistent morphology thicknesses between strains. Relative strain dependent changes in photoreceptor thickness can be observed in BLE housed mice **(F)**. Abbreviations: outer nuclear layer (ONL), outer limiting membrane (OLM), photoreceptor inner segment (IS), photoreceptor outer segments (OS), retinal pigment epithelium (RPE), Bruch’s membrane (BM), retinal pigment epithelium-Bruch’s membrane complex (RPEBM), and choriocapillaris (CC).

Upon reviewing SD-OCT mouse data from these experiments, we noticed that a region of the photoreceptor outer segments underwent a dynamic change in reflectivity in two of the three strains examined following relocation from dim to bright lighting conditions (Figure 3A). When analyzed by using LRP, amplitude changes to the outer segments were appreciable for BALB/c and B6-albino mice (Figures 3B–D; OS box-up arrows) but not B6 mice (Figure 3D; OS box). Also notable was the repositioning of the RPE/BM which suggested that outer segment thickness was another important metric to ascertain (Figures 3B–D; BM-arrows).

LRPs grouped by dim (Figure 3E) and bright (Figure 3F) conditions demonstrate the relative similarities and differences in photoreceptor morphology between the three mouse strains. In Figure 3E under DLE, the two albino strains show differences in reflective amplitude relative to the B6 mouse at the distal OS, RPE, BM and choroid locations predominately due to the lack of melanin pigment in the RPE and choroid. Nevertheless, Figure 3E reveals that LRPs of the photoreceptor morphological features are still similar between strains and can be co-aligned and identified using this method of comparison. Figure 3F demonstrates the changes that occurred following one week of BLE. Most notable are the changes in the minima from the proximal, or basal aspect, of the photoreceptor OS in the two albino mice and the shift in the location of the RPE/BM by the shortening or elongation of the OS relative to the pigmented B6 mouse. An analysis of these changes in a small cohort of mice is shown in Figure 3G using the OS signal slope between the distal to proximal, or apical to basal, aspects of the OS reveal that both albino strains show significant differences in this metric relative to B6 mice maintained under DLE versus 1 week of BLE.

### 3.3 Analyses 4–6: Dynamics of photoreceptor layer reflective signal amplitude

**FIGURE 4:**
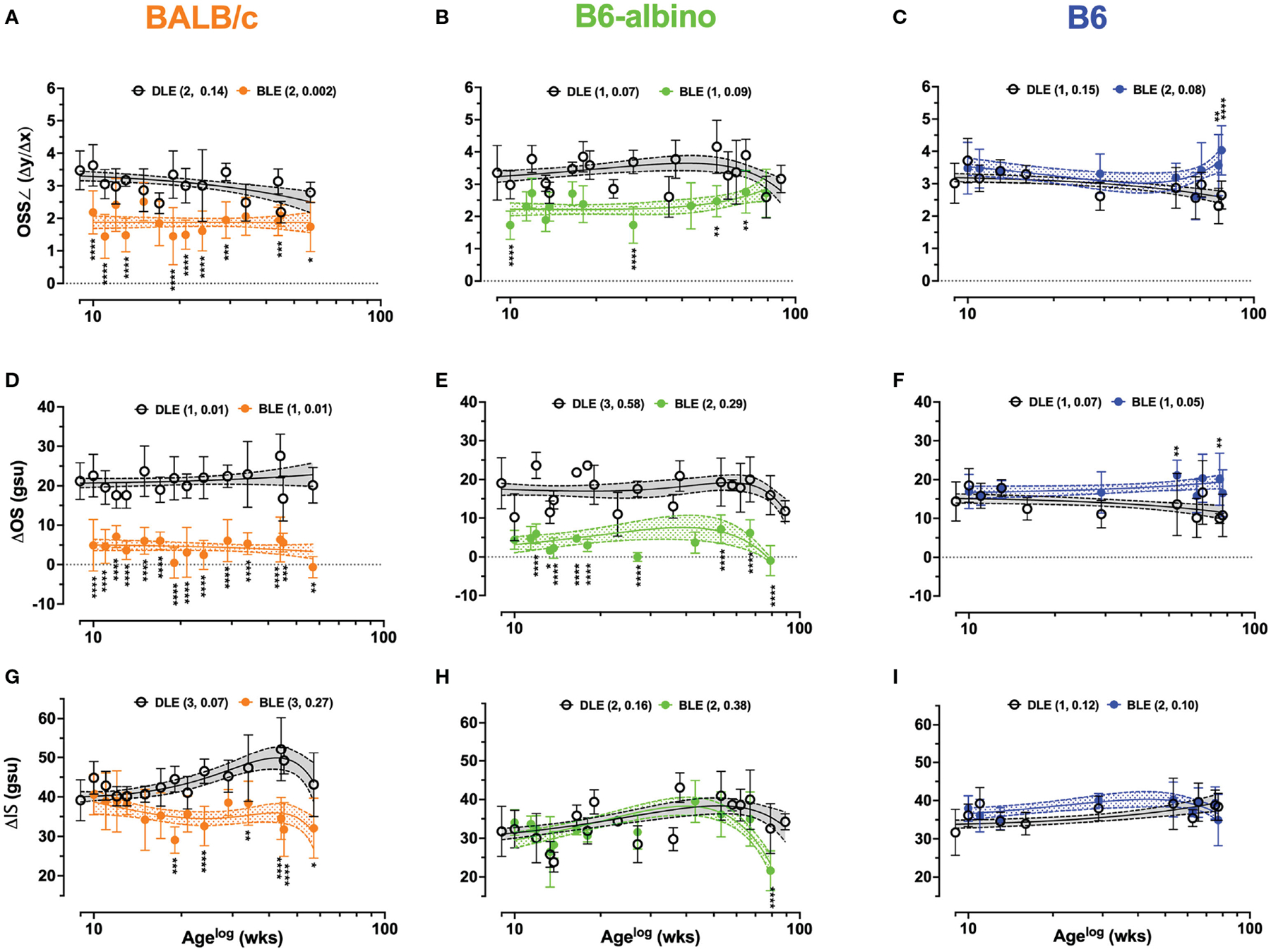
Dynamic trends extracted from LRP data for Analyses #4–6 in BALB/c, B6-albino, and B6 mouse strains. Trends between DLE vs. BLE groups are displayed for: Analysis #4 of the OS signal slope (OSS∠) **(A**–**C)**, Analysis #5 of the ΔOS **(D**–**F)**, and Analysis #6 of the ΔIS **(G**–**I)**. Datapoints show mean+/−SD and curve fit are mean+/−95% CI. Legends indicate the curve fit polynomial order and R-squared in parenthesis for both DLE and BLE trends.

Predominant features in SD-OCT images, also referred to as bright and dark reflective bands, were revealed using the LRP analysis and assessed for changes in relative signal amplitude or signal slope as previously described (Table 2; Analyses #4–6). Figure 4 shows the results from all three strains investigated and includes Analysis #4 the OS signal slope and Analysis #’s 5 & 6, which is the absolute difference between the OS and IS signal amplitude minima and the IS-OS junction maxima, respectively.

Figures 4A–C shows the results for Analysis #4, the OS signal slope (OSS∠). Baseline OSS∠ was similar for BALB/c and B6-albino being 3.4 ± 0.59 and 3.5 ± 0.85 au, respectively. Due to pigmented RPE and choroid, B6 mice registered (3.0± 0.6 au) a significantly lower OSS∠ at baseline than BALB/c (p=.0097), but not B6-albino (p=.0528). After switching from DLE to BLE post-baseline, OSS∠ measures from one-week post showed an abrupt, significant decrease to 2.2 ± 0.66 and 1.7 ± 0.44 au for BALB/c (Mean Diff. = 1.3; p=.0001) and B6-albino strains (Mean Diff. = 1.6; p<.0001) and an insignificant, half-unit increase for B6 (Mean Diff. = 0.468; p=.1189). This trend was largely sustained throughout the duration of the experiment timeline with exception to B6 and B6-albino which showed some deviation towards the end of the monitoring period. In Figures 4A, B, BALB/c and B6-albino consistently displayed a propensity for reduced OSS∠ in BLE vs. DLE groups. OSS∠ was significant between BLE vs. DLE treatment groups in 64% (9/14) and 40% (4/10) of BALB/c and B6-albino synchronous imaging timepoints, respectively. As B6-albino mice approached 80 weeks of age, OSS∠ trends between BLE and DLE groups converge in relationship to the rapidly decreasing changes in RIF/AFF count and ONL thickness (Figures 1H, Q, Z). In contrast, B6 BLE and DLE groups diverge after 70 weeks and became significant at 80 weeks (Figures 4C, F), indicating some propensity for inducing chronic light-induced changes similar to that found with the AFF measures for B6 (Figure 1R).

The trends observed in Analysis 4 are further reinforced by Analysis 5 results shown in Figures 4D–F, which revealed more appreciable and significant changes to the ΔOS than OSS∠ for both BALB/c and B6-albino, relative to the B6. Mean ΔOS measures at baseline was similar for BALB/c and B6-albino (Mean = 21.2 ± 4.7 & 19± 6.6 gsu), but significantly different for B6 (Mean~14.4± 5.1 gsu; p<.0001 vs. BALB/c & p=.0003 vs. B6-albino). After switching from DLE to BLE post-baseline, ΔOS measures from one-week post significantly decreased in BALB/c (Mean Diff. = 16 gsu; p<.0001) and B6-albino strains (Mean Diff. = 14 gsu; p<.0001) and increased insignificantly for B6 (Mean Diff. = 2.56 gsu). Similar to OSS∠, these trends were sustained with exception to B6 and B6-albino strains, which deviated at the end of the monitoring period. Qualitatively for BALB/c, ΔOS trends less variable and more consistent than OSS∠ trends over the experiment duration. In Figures 4D–F, BALB/c and B6-albino consistently displayed reduced ΔOS measures in BLE vs. DLE groups. ΔOS was significant between BLE vs. DLE treatment groups in 100% (14/14) and 90% (9/10) of BALB/c and B6-albino synchronous imaging timepoints, respectively. In B6, significant changes to ΔOS trend materialized and were significant at ages of 55 and 80 weeks for BLE exposures of ~46 and 71 wks. These observations suggest that ΔOS Analysis 5 may be better than the OSS∠ Analysis 4 for quantifying BLE vs. DLE induced morphological changes to the photoreceptor outer segments of mice.

Following the successful use of both the OSS∠ and ΔOS, ΔIS Analysis 5 was performed to elucidate photoreceptor inner segments trends as displayed in Figures 4G–I. At baseline, BALB/c showed a significantly different ΔIS mean of 39 gsu relative to ~32 gsu for both B6-albino and B6 (p<.0001), which highlights an interesting difference in inner segment reflectivity between BALB/c and B6-albino and pigmented strains. No significant changes to ΔIS are recorded 1-week post-baseline after switching the mouse strains from DLE to BLE. Long-term trends recorded for B6-albino and B6 were largely unchanged, with exception to a sudden, significant decrease (Figure 4H; p<.0001) in B6-albino ΔIS measures at 80 weeks of age that coincided with rapid decreases in RIF/AIF count and ONL thickness (Figures 1H, Q, Z). BALB/c ΔIS measure trends between BLE and DLE diverged at ~6 wks post-baseline and at ten weeks post-baseline (age 19 wks), became significant and persisted for the remainder of the study (Figure 4G; DLE vs. BLE). During this time, 75% (6/8) of synchronous BLE ΔIS measures were significant relative to the DLE.

### 3.4 Analysis 7: Dynamics of photoreceptor layer reflective morphology

**FIGURE 5:**
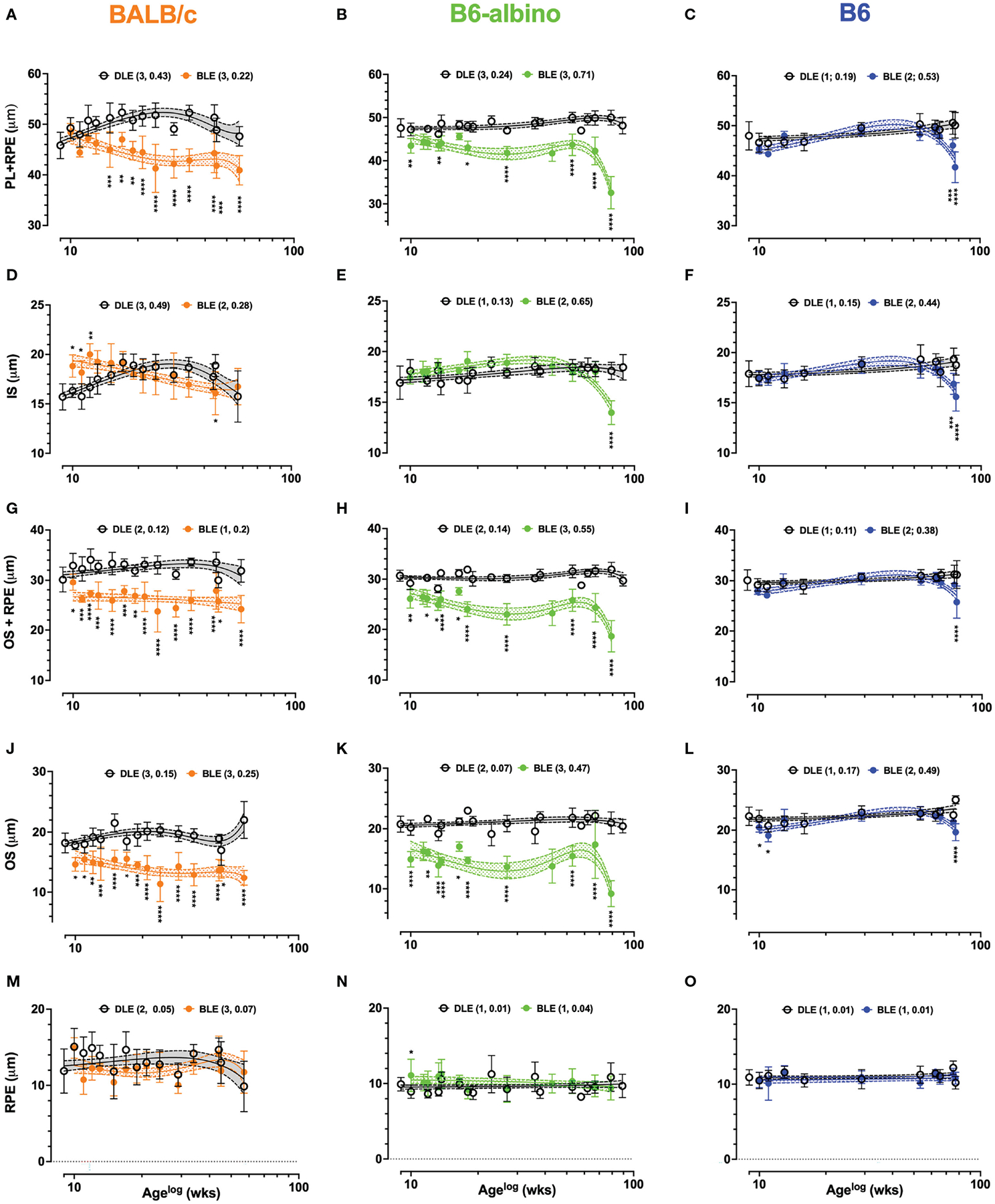
Dynamic trends in lamina thicknesses measured and extracted from LRP data in BALB/c **(A, D, G, J, M)** B6-albino **(B, E, H, K, N)** and B6 **(C, F, I, L, O)** mouse strains. Each graph shows long-term trends comparing DLE and BLE groups for PL+RPE (Analysis #7; **A**–**C**), IS (Analysis #7a; **(D**–**F)**, OS+RPE (Analysis #7b; **G**–**I**), OS (Analysis 7b.1; **J**–**L**) and RPE (Analysis #7b.2; **M**–**O**). Datapoints show mean+/−SD and curve fit are mean+/−95% CI. Legends indicate the curve fit polynomial order and R-squared in parenthesis for both DLE and BLE trends.

Substantial changes observed in Analyses 1–6 warranted additional investigations in photoreceptor laminar thickness using the approach described in Analysis 7 of Table 2. An initial pass at performing Analysis 7 simply included measuring the photoreceptor layer (PL) from OLM to either BM for both albino strains, or alternatively, to the choriocapillaris (CC) in B6. The results of the PL + RPE measures are shown in Figures 5A–C. At baseline, PL+ RPE was 45.8 μm for BALB/c, which was significantly different from both B6-albino (47.6 μm; p = .0045) and B6 (48 μm; p =.0006). A week after switching from DLE to BLE, the PL+RPE significantly thickened by 2.6 μm in BALB/c (48.4; p=.045) relative to baseline and significantly thinned by 4.2 μm and 2.6 μm in B6-albino (43.5 μm; p =.0004) and B6 (45.4 μm; p=.003), respectively.

In BALB/c (Figure 5A), PL+RPE measures in the BLE group decreased following baseline, became significantly different at 6 wks post-treatment onset, and remained significant for the duration of the study in all (100%; 10/10) of the possible synchronous measurement timepoints occurring thereafter. PL+RPE measures of the BALB/c DLE group showed a tendency to increase with age until ~30 wks of age, then slowly decreasing thereafter. Somewhat similar to BALB/c, the B6-albino BLE trend immediately separated and could be delineated from the DLE group trend for the entire study duration (Figure 5B). However, instead of exhibiting a precipitous decline in BLE PL+RPE thickness like BALB/c, B6-albino showed an immediate and significant decrease at 1 wk post-baseline that turned into a sustained difference through 56 wks of BLE (Age 65 wks) until a rapid-onset decrease was observed at 71 wks (Age 80 wks). Seventy percent (7/10) of synchronous PL+RPE measures were significant between BLE vs. DLE groups. In Figure 5C, PL+RPE measures in B6 mice showed little change over the study duration with exception to the last two timepoints where the trend significantly decreased at ages 75 & 77 wks following 66 & 68 wks of BLE exposure.

A second pass through the LRP data, referred to as Analyses 7A & 7B in Table 2, resulted in separating the PL+RPE data into individual inner and outer segments + RPE components. This pass was conducted to more precisely isolate which photoreceptor lamina(s) was affected by BLE. The results for Analyses 7A (IS) and 7B (OS+RPE) are shown by Figures 5D–F and 5G–I, respectively. IS thickness at baseline was significantly different between all strains (p<.0001). Baseline IS thickness was 15.7 ± 1.3, 16.9 ± 1.6 and 17.9 ± 1.3 μm for BALB/c, B6-albino, and B6 strains, respectively. One-week following BLE, BALB/c IS significantly increased in thickness to 18.8 ± 1.1μm (p<.0001) whereas B6-albino and B6 did not significantly change (Figures 5D vs. E–F). In Figure 5D showing the BALB/c data, IS measures from the BLE group remained thicker, or perhaps elongated relative to the DLE group for about 6–8 wks post-baseline. Three of the six initial synchronous measures collected between BLE and DLE groups were significantly different for 1, 2 & 3 wks post-baseline as shown in Figure 5D. IS thickness for the BLE group decreased with experiment duration and crossed over the DLE group at 10 wks post-baseline as the former was decaying and the latter increasing. Despite the diverging dynamics between BALB/c BLE and DLE, both groups ended up at the same IS thickness after 70+ wks of exposure which coincidentally, was not significantly different from baseline. B6-albino trends between BLE and DLE, shown in Figure 5E, were relatively similar until the end of the study period when the BLE group significantly decreased on the last time point after 70+ weeks of exposure. The B6 strain showed a similar response as the B6-albino (Figures 5E vs. F).

As shown in Figures 5G–I, the OS+RPE information is complimentary to the IS data shown in Figures 5D–F. There were no significant differences in OS+RPE thickness at baseline between mouse strains with all measuring ~30 μm. One-week post-transfer to BLE, BALB/c (26 μm; p<.0001) and B6-albino (27.9 μm; p=.0014) strains registered a significant decrease in OS+RPE thickness. Following 1-week of BLE, B6 did not change significantly from baseline. At 2-wks post, BALB/c showed a significant and sustained decrease in OS+RPE thickness for the remainder of the study duration with all (14/14) of the possible synchronous time points being significant from the DLE group (Figure 5G). Similar to BALB/c is B6-albino (Figure 5H), in which the OS+RPE of the BLE group had a propensity to be shortened in all (10/10) of the possible synchronous time points. Notable again is that the OS+RPE thickness rapidly decreases after 70 weeks of BLE. In contrast, the B6 BLE group does not drastically differ from their DLE counterparts over the study duration with exception to the last data point collected after 68 wks of exposure which showed a significant decrease in BLE vs DLE (Figure 5I).

Following the interesting observations obtained from Analyses 7a & 7b, it seemed prudent to partition out the OS (Figures 5J–L) and RPE (Figures 5M–O) laminar information with Analyses 7b.1 and 7b.2. In Figures 5J–L, OS thickness was significantly different (p<.0001) at baseline between BALB/c (18.2 ± 1.6 μm), B6-albino (20.8 ± 1.3 μm), and B6 (22.3 ± 1.5 μm) strains. One-week post-transfer from DLE to BLE, all three strains exhibited a significant decrease (p<.0001) in OS thickness relative to baseline. OS thicknesses in BALB/c, B6-albino and B6 decreased by 3.5, 5.8 and 1.8 μm, respectively. For BALB/c and B6-albino, OS thickness in BLE groups remained significantly suppressed for the study duration relative to DLE groups (Figures 5J, K). All synchronous measurements between BLE and DLE groups of both BALB/c and B6-albino strains were significant indicating a persistent thinning of the photoreceptor OS from chronic BLE (Figures 5J, K). After 70+ wks of BLE, B6-albino OS showed a rapid decrease in thickness at 80 wks of age, similar to that found for ONL, PL, IS and OS+RPE evaluations. In Figure 5L, B6 showed two initial instances of significant reductions in OS thickness induced by switching from DLE to BLE. This observation resolved by 4 wks post-baseline and remained until the last timepoints of the study in which a significant decrease was observed in the BLE vs. DLE group (Figure 5L; BLE trend @ age ~77 wks). Contrary to the dynamic changes observed in the OS with Analysis 7b.1, RPE measures obtained in Analysis 7b.2 are more subdued as shown in Figures 5M–O. RPE thickness was significantly different (p<.0001) at baseline between BALB/c (11.9 ± 3.0 μm), B6-albino (9.9 ± 0.9 μm), and B6 (10.9 ± 1.0 μm) strains. One-week post-transfer from DLE to BLE, only BALB/c exhibited a significant decrease (3 μm; p=.0004) in RPE thickness relative to baseline. With exception to one synchronous datapoint in the B6-albino strain, no significant differences were registered for RPE thickness between BLE and DLE groups of each strain (Figures 5M–O).

### 3.5 Short-term exposure testing for determining whether light-induced outer segment changes are permanent or reversible

**FIGURE 6:**
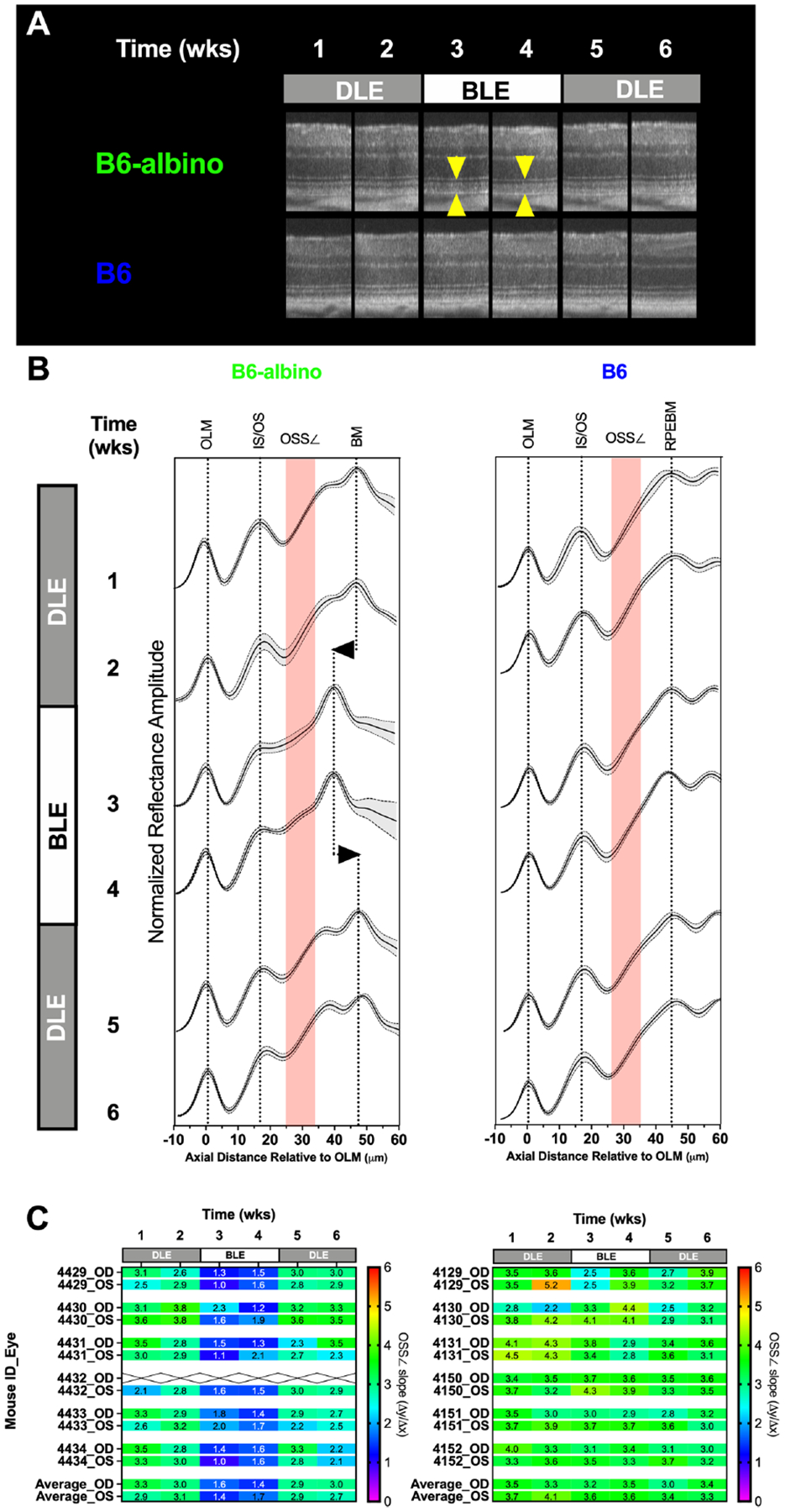
Short-term DLE-BLE-DLE exposure testing results demonstrate that light-induced photoreceptor layer outer segment changes is a non-permanent, reversible biomarker in B6-albino mice relative to B6 which are not responsive to this insult. **(A)** Representative SD-OCT images from a B6-albino mouse retina showing the retina before (DLE; wks 1–2), during (BLE; wks 3–4) and after (DLE; wks 5–6) light susceptibility challenge. Yellow arrows indicate the photoreceptor layer, the region of the outer retina responsible for light absorption and phototransduction which is highly affected by BLE. Arrows bracket the region of increased OCT signal back-reflectivity and reduction in photoreceptor layer lamina thickness. By comparison, B6 mice are unaffected by this challenge as qualitatively no alterations to the photoreceptor layer can be observed. **(B)** Averaged LRPs showing the Mean ± 95%CI or both BALB/c and B6-albino are shown for each consecutive the mice were imaged under DLE-BLE-DLE conditions. The regions where the OSS∠ (Δy/Δx) is measured is denoted by the red shaded area. The change in signal back-reflection and shortened photoreceptor layer thickness, as revealed by the shift in BM, is easily discerned at weeks 3 &4 for B6-albino. In comparison, averaged LRPs from B6 mice show no changes in signal back-reflectivity or movement of the RPEBM. **(C)** A heat-map of individual mice (OD & OS) showing the mean OSS∠ values averaged from temporal, nasal, superior and inferior regions. B6-albino OSS∠ are reduced by ~2 (50%) slope units within one week of being switched from DLE to BLE. This reduction in slope was very significant (Adj. P<0.0001) for BLE at both weeks 3 & 4. B6 mice as a group showed a decreasing trend in the average OSS∠ but this change was not statistically significant.

Results from the DLE-BLE-DLE experiment are shown in Figure 6. B6 and B6-albino cohorts subjected to alternating light exposure conditions on a bi-monthly basis for 6 weeks exhibited different outcomes. Figure 6A shows representative morphological differences in the reflective signal obtained from the temporal retinal region of a B6-albino vs. B6 mice. Reflective morphology in the B6 strain remained similar through the 6-week progression. In contrast, the B6-albino example shown reveals a similar photoreceptor layer morphology collected during the initial, first two weeks of DLE (Wk’s 1 & 2), followed by a robust change the next two consecutive weeks under BLE (Wk’s 3 & 4; yellow arrows). The robust change observed abruptly resolves upon returning the animal to the dim vivarium for the final two weeks under low illuminance conditions (Wk’s 5 & 6).

To better accentuate these changes, individual LRPs from the Superior, Nasal, Temporal and Inferior regions were averaged to generate mouse cohort averages as shown in Figure 6B. LRP means with +/− standard deviations are shown for each weekly imaging timepoint in conjunction with a region of interest indicating where the OS signal slope is measured (Figure 6B; red shaded boxes). Following a switch from DLE to BLE, the OS minima is moderated (left edge of the red shaded box) in B6-albino, but not B6 mice. Alteration of the OS signal slope by the change in OS minimum can be qualitatively observed at Wk’s 3 & 4 for B6-albino relative to Wk’s 1 & 2. Also noted in B6-albino is a displacement of the RPEBM due to the reduction in OS thickness (Figure 6B; vertical dotted lines with left/right arrows). Upon returning B6-albino mice to DLE the moderated OS minima, change in signal slope, and shortening of OS thickness all resolved within one week. In contrast, no similar changes were observed in the B6 cohort subjected to the same treatment challenge.

Figure 6C shows a heat map of changes in OS signal slope (OSS∠) recorded for each individual mouse eye that underwent the DLE-BLE-DLE challenge. The OS signal slope results are displayed from 0 to 6 slope units with a color scale bar. For B6-albino mice, a consistent signal slope (green) is observed during the first two weeks under DLE conditions, which abruptly swings to lower slope values (blue) following BLE and is restored following the switch back to DLE. In contrast, the B6 cohort does report a few of instances of variability, but predominantly averages between 3 and 4 slope units (green) throughout the challenge duration. Supplemental Figure S5 shows a dot plot of the OS signal slope vs challenge duration for these two mouse strains. Initially, B6-albino mice showed a mean signal slope of ~3 whereas the B6 were higher at ~3.7, which can be attributed to the presence or absence of RPE melanin pigment. Initial measures remained unchanged for both strains following an additional week of DLE. After switching to BLE, the OS signal slope in the B6-albino was significantly reduced by a factor of two (~3 vs. ~1.5) whereas B6 were unaffected by this change. Upon returning the mice to DLE conditions, the changes previously observed in B6-albino rebounded significantly one week later and remained for the final evaluation time point. B6 that were unaffected relative to B6-albino mice, did seem to show a mild decreasing trend in OS signal slope over the experimental duration but these changes were not significant relative to the baseline time point at week 1.

### 3.6 Spearmen’s ρ correlation test and predictive power score matrices

We tested whether any of the optophysiological imaging biomarkers observed could be correlated to, or predictive of, impending photoreceptor degeneration. For each mouse strain, cross-correlation matrices (shown in Supplemental Figure S6) were obtained using nonparametric Spearman’s and Predictive Power Score tests. An advantage of the Spearman’s correlation is that it shows both positive and negative correlations, however, a limitation is that it is incapable of revealing any bi-directional asymmetry or non-linearity between variables for a particular outcome of interest. As observed in Supplemental Figure S6, the Spearman’s correlation matrices are information busy datasets and in contrast, the PPS matrices simplified the data visualization and accentuated focus for the strongest relationships between variables, or “targets” and “fields”, as they are referred to in PPS.

**FIGURE 7:**
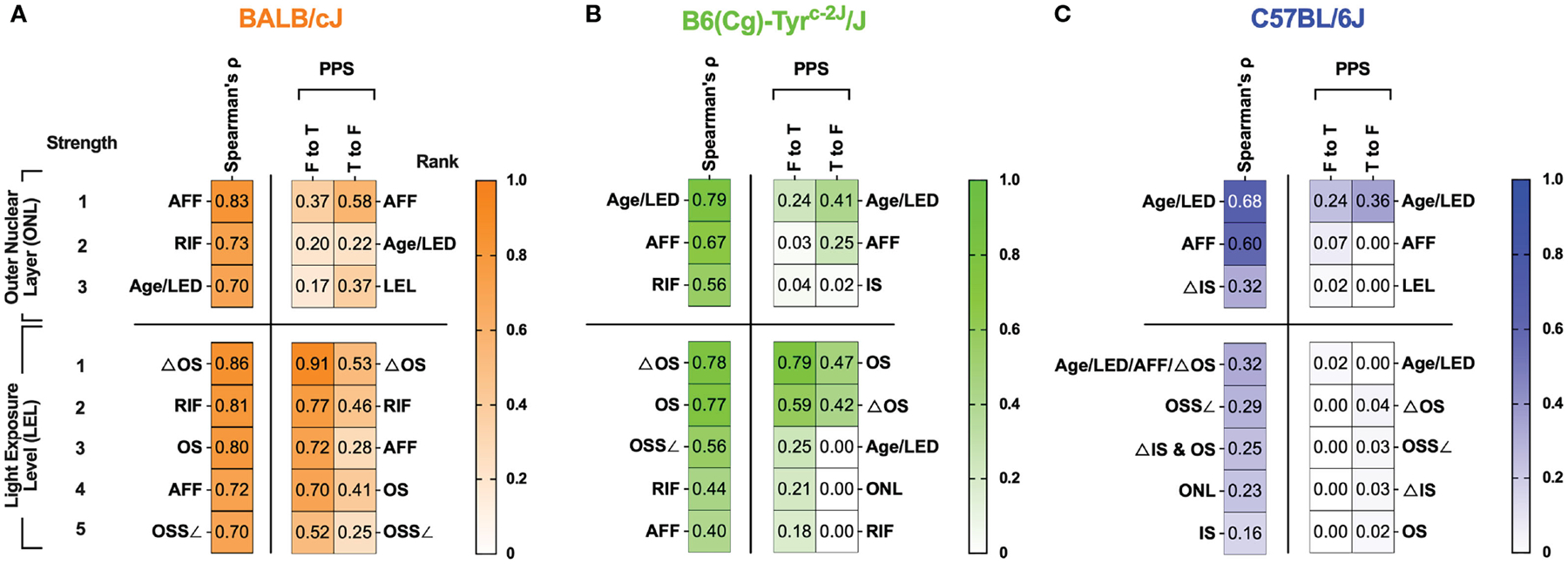
Summarized results of Spearman’s cross-correlation and PPS matrices related to Outer Nuclear Layer (ONL) thickness and Light Exposure Level (LEL) for BALB/c **(A)**, B6-albino **(B)** and B6 **(C)** mouse strains. Are any of the biomarkers capable of predicting retinal degeneration? For BALB/c mice, the best indicator for anticipating ONL thickness was AFF count. In both B6-albino and B6, animal Age and Light Exposure Duration (LED) was the best indicator of ONL thickness followed closely by AFF count. Thus, as an imaging biomarker, AFF was clearly the best indicator for determining whether ONL degeneration could be anticipated in a mouse eye. Are any of the biomarkers capable of revealing light-stress in an animal? For BALB/c and B6-albino mice, changes in the photoreceptor proximal outer segments observed by SD-OCT imaging are an excellent indicator of LEL, or light stress, in the vulnerable albino strains. ΔOS, which is simply the contrast between the proximal outer segments and the IS-OS transition zone appears to be the best method for indicating an animal under light stress from ~150–200 lx in both albino strains. Interestingly, this analysis has shown that ΔOS is an improvement over our original approach of using the outer segment signal slope (OSS∠) as the best method to quantify this alteration in reflected signal.

Summarized findings related to ONL thickness and light exposure level (LEL) are shown in Figure 7 where the strongest relationships have been ranked in descending order. The strongest PPS ranks were typically similar to the strongest Spearman’s ranks. Nevertheless, PPS was helpful in showing that some correlations were weaker than reported by correlation due to the various and variable trends in biomarker dynamics. For example, AFF in BALB/c mice correlated very strongly (0.83) with ONL thickness. However, the PPS analysis revealed that the strength of this correlation is not as anticipated as PPS values indicated weak [0.37 (F to T)] and moderated [0.58 (T to F)] values below that reported by Spearman’s (0.83). A better example is RIF, which our group has demonstrated develop prior to the appearance of AFF in the BALB/c mice under BLE conditions (Bell et al., (5)). With this biomarker, Spearman’s shows that there is a strong (0.73) relationship between RIF and ONL thickness, which in terms of correlation strength, ranks second only behind AFF. In contrast the PPS analysis for RIF to ONL was very weak [0.00 (F to T)] and weak for ONL to RIF [0.39 (T to F)]. As a result of the PPS analysis, RIF does not even rank in the top three for predicting ONL degeneration.

A comparison of the graphical trends for RIF and ONL in Figures 1G, Y, respectively, sheds light on the disconnect between these two biomarkers as RIF are changing in a non-linear manner (e.g. increasing, peaking, falling) over time while ONL degeneration is decreasing steadily at a near constant rate. In addition, comparing Figures 1P, Y shows why AFF in this particular dataset, is better at predicting ONL degeneration than RIF because AFF progression increases linearly for most of the observation period and subsequently plateaus without further changes. As a result, dynamic trends between AFF and ONL match more closely during the period of evaluation and are the best predictor of ONL degeneration rather than RIF. As these two examples demonstrate, PPS has revealed more detail about the comprehensive biomarker data and slightly reduced the complex nature of the correlation matrix interpretations.

An important question we had while undertaking this study is whether any of identified biomarkers could be indicative, or even better, predictive of photoreceptor degeneration. The summary table in Figure 7 helps demonstrate which biomarkers performed best for correlating with and predicting ONL and LEL. In BALB/c, AFF, RIF and Age/Light exposure Duration (LED) exhibited strong to very strong correlations (0.7–0.83) for ONL degeneration for AFF (0.83), RIF (0.73), and Age/LED (0.70). The PPS analysis was not as supportive of the strength of these correlations for AFF as it was shown to be a weak ([0.37 (F to T)] to moderate [0.58 (T to F)] predictor of ONL thickness, nevertheless, it was the strongest predictor by rank among the available biomarkers. Like BALB/c but falling in different order, biomarkers found correlating with ONL for B6-albino in order of strongest to weakest, was Age/LED (0.79), AFF (0.67), and RIF (0.56). Again, PPS analysis did not support the strength of the correlations as all predictions were weak or very weak. Nevertheless, Age/LED was found to be the strongest predictor and best correlative indicator of ONL thickness in B6-albino. B6 were similar to B6-albino in that Age/LED (0.68) and AFF (0.60) again provided the strongest correlations for ONL thickness. The PPS analysis also showed that these two correlations were weakly and very weakly predictive of ONL thickness. Similar to B6-albino, Age/LED was found to be both the strongest predictor and best correlative indicator for ONL thickness in B6.

Secondarily, we decided to determine whether biomarkers could be predictive of LEL or light stress in these strains. Examining the BALB/c data presented in Figure 7, several cSLO and SD-OCT biomarkers correlated very strongly and strongly with LEL including ΔOS (0.86), RIF (0.81), OS (0.80), AFF (0.72), and OSS∠ (0.70). In this case, PPS predictions generally supported the correlations but in a slightly rearranged order. This comparison demonstrates that each these biomarkers are moderate to strong indicators of light exposure levels or light stress in BALB/c mice with the clear winner being ΔOS [0.91 (F to T)] & [0.53 (T to F)]. If one recalls, ΔOS is the change in signal reflectivity within the proximal outer segments relative to the IS-OS transition; in essence an altered signal contrast in the photoreceptor layer observed by SD-OCT imaging. Ranking second to ΔOS was RIF which is observed by cSLO imaging instead of SD-OCT, an additional manner to confirm light stress using another imaging modality. In the B6-albino, biomarkers correlating with LEL were similar to those observed in BALB/c but differed in correlation strength. Strong correlations were observed for ΔOS (0.78) and OS (0.77). Among all the biomarker data collected, only ones related to the photoreceptor outer segments delineated by SD-OCT were found to be strongly or moderately predictive of LEL and this includes the OS [0.79 (F to T)] & [0.47 (T to F)] and ΔOS [0.59 (F to T)] & [0.42 (T to F)] biomarkers, respectively. In contrast to both albino stains, pigmented B6 mice had no biomarkers that could be correlated to or predictive of LEL.

## 4 Discussion

In this study variable lighting conditions commonly encountered within small animal vivaria were exploited to chronically expose popular wildtype strains of laboratory mice over an extended period. Mice were exposed to dim and relatively bright intracage illuminance levels mimicking bottom and top shelf cage rack positions, respectively. Mice underwent multiple non-invasive fundus imaging sessions to periodically catalog dynamic changes resulting from seemingly innocuous levels, as perceived by normal human vision, of visible light exposure. In mammals with pigmented irids, the illuminance levels employed (~150 lx) do not typically result in adverse effects unless the animal subject exhibits a genetic or biological susceptibility to phototoxicity. However, as demonstrated, mice exposed to even these low to moderate illuminance levels can be detrimental if allowed to compound over time, whereby exerting long-term photoreceptor stress and cellular damage to the retina of albino mouse strains.

Photoreceptor toxicity has been previously reported in multiple albino and pigmented strains and between various genetic crosses in an attempt to isolate the underlying causes of light-induced retinal degeneration (19–21, 26–28). LaVail et al. demonstrated that B6-albino are remarkably resistant to light damage relative to BALB/c and they suspected the difference is attributed to an unknown genetic mutation (20). In the years following many vision scientists have put forth considerable effort to determine the cause and effect of phototoxicity in albino rodents. From these works, an array of genetic, biochemical, and anatomical rationales has been proposed as possible contributors to light-induced retinal degeneration in BALB/c and B6-albino mice. A less than exhaustive list of these possibilities includes: (1) amino acid substitutions like Leucine-to Methionine variant at Codon 450 for the RPE65 protein (e.g. RPE65-Met450 vs. RPE65-Leu450) (19, 29), (2) RPE65 protein expression levels (28) (3) rhodopsin recycling and regeneration rates (28, 30–33), and the (4) lack of dark melanin pigment and its influence on the number of photons reaching the retina (31, 34).

Previous works determined that both the slower recovery from photobleach and lower gain of activation characteristic of the B6-albino strain may contribute to the mechanism by which it is protected from light-induced photoreceptor death relative to BALB/c (30). To this day the overarching consensus is that light susceptibility and phototoxicity is related to genetic variant and associated rhodopsin-mediated mechanisms of these variants (26, 35, 36). A notable limitation of almost all previous studies is the lack of long-term, temporal data for characterizing the dynamic changes related to light dosimetry. Also, many studies conducted previously were performed before non-invasive ocular imaging was widely available and thus comprehensive evaluations of retinal dynamics relating to exposure duration and intensity are sparse. In contrast to our experiments, most light exposure or “light challenge” experiments involve short-term, high lux (>3–10klx), acute, continuous light exposure ranging from hours to a few weeks. These short-term acute experiments do not represent typical daily cyclic (light:dark) exposures and thus our vivaria experiments appear to effectively expose mice in a familiar environment free of stress. Reducing stress is important as it has been shown to produce protective glucocorticoids and neurotrophic factors that can confound acute light exposure outcomes by making animals more resistant to damage (29).

Not unlike previous studies showing histological and biochemical changes under similar light exposure levels, this study reinforced existing imaging phenotypes including the development of AFF, photoreceptor degeneration, outer segment shortening and changes in outer retina reflectivity (5, 26, 37–41). Presented here is evidence for a new visual and quantifiable imaging biomarker (i.e., the changes to the proximal photoreceptor outer segments) related to light stress. Overall, this work demonstrated multiple ways one can visually detect light stress-induced retinal changes from cSLO or SD-OCT images and analyze these changes to reveal existing and newly presented correlative measurements. For cSLO, we determined that the best biomarker was AFF and for SD-OCT it was ΔOS, which was the change in proximal OS relative to the IS-OS transition.

As previously reported, we found that B6-albino mice undergoing a light stress challenge were indeed protective, or less susceptible to light damage. For many weeks following to BLE, B6-albino did not show significant retinal changes before displaying, in contrast to all previous assessments of this strain, an appreciable decline in ONL thickness (Figures 1Z and 2F). Unfortunately, this unexpected and surprising downward trend in ONL thickness was only observed once as the study was discontinued soon thereafter. Nevertheless, the last datapoint contains pooled data from two independent progeny of B6-albino mice born to different parents ~1.5 months apart. Both litters experienced the same vivaria conditions and were imaged a total of three times throughout the study, thus experiencing similar, consistent parameters. On the very last imaging session, conducted 1.5 months apart between the two groups, mice from the 1^st^ litter had an average ONL thickness (mean+/−SD) of 33+/−1.39 μm and the 2^nd^ litter 29.8+/−4.26 μm, which was not statistically different (Unpaired Two-tailed t-test; p=0.1048). Thus, with two independent litters of mice we have provided additional evidence that this downward inflection of ONL thickness in B6-albino mice does not appear to a serendipitous occurrence. One additional detail in support of this observation is that the sudden change in ONL thickness coincides with observed downward trends in RIF and AFF biomarkers as well (Figure 2B). To our knowledge this is a novel characteristic to report for the B6-albino strain.

In BALB/c, the ONL degenerated at a consistent, but eventually slowing rate (i.e. as if approaching an asymptote; Figures 1Y and 2A), from BLE onset while B6-albino experienced a delayed degeneration onset only after RIF and AFF reached peak inflection points. Following reaching these maxima, all three biomarkers, RIF, AFF and ONL, began to decrease in unison for B6-albino. Accommodative changes in the susceptibility of light photoxicity has been previously documented in BALB/c as Kaldi et al., (42) demonstrated that mice reared under dim (5 lx) vs. bright (400 lx) cyclic light were more susceptible to a continuous 3-day light exposure challenge of 3000 lx (42). Referred to as “light preconditioning”, this unique characteristic exhibited by BALB/c has been exploited in other studies to investigate retinal neuroprotection and up- or down-regulation of associated neuroprotective following bright light challenge (43, 44). As a result of these previous studies, it’s not an extrapolation to believe that these rate changes in ONL degeneration of BALB/c, whether steady, increasing or decreasing, are due in part from the biology of the retina altering its response to BLE insult.

In comparison, B6 mice were completely absent of RIF and did not develop ONL degeneration. They did, however, accumulate more AFF over time and this finding was particularly interesting as mice in the BLE group developed significantly more AFF than their DLE counterparts. During this same time, OSS∠ and ΔOS trends (Figures 4C, F) in the B6 BLE group increased concomitantly with increasing AFF count (Figure 1R), suggested a relationship between these biomarkers for B6 mice. This implies that the processes involving recruitment of AFF, or the presence of AFF themselves, contribute to increase sub-retinal space reflectivity relative to the proximal OS whereby increasing the OSS∠ by one slope unit from ~3 to 4. This could be explained by inflammatory cells transitioning from a ramified to active state, becoming engorged with cleaved photoreceptor outer segments and cellular material, increasing in cross-sectional diameter and SD-OCT reflectivity relative to the proximal OS region. This was opposite of what was observed in the albino mice in which OSS∠ and ΔOS trends were altered after only one week of being relocated from DLE to BLE conditions and RIF and AFF, relatively speaking, increased at a slower rate over many weeks. The change in OSS∠ and ΔOS in the proximal photoreceptor outer segments in albino mice is very different than B6 and likely due to visual cycle dysfunction and compromised visual pigment-regeneration of retinyl esters in albino animals. The presence of AFF and their increase with age has been documented by others (5, 37, 45–47), but a difference in AFF count due to vivarium light exposure level has not been previously reported in B6 mice to our knowledge. These data suggest that B6 mice may be sensitive to long-term light stress but remain subthreshold for photoreceptor degeneration at the illuminance levels employed and the age range investigated. Clearly through the data presented here with multiple biomarkers, albino mice are highly susceptible to vivarium light exposure and signal that sensitivity with ease using non-invasive imaging.

Melanin acts as a cellular antioxidant and is supposed to protect RPE cells by scavenging free radicals, quenching electronically excited states, and sequestering redox-active metal ions elicited by visible light or by redox-active metal ions (48–53). Also, the human RPE melanosomes in cells irradiated with visible light have been reported to result in photobleaching (52). Our previous work analyzing melanin in cryosections from AMD donors (54) and a manuscript analyzing mice suggest that melanin near-infrared AF is a consequence of photic and oxidative stress (55).

Qualitatively, when collecting IR- and BAF-cSLO images of the fundus in BALB/c and B6-albino, we observed RIF developing in a different geographical manner over time between strains. BALB/c developed RIF in a random manner over the visible fundus area whereas B6-albino developed central imaging FOV and then appeared to propagate radially outwards to the periphery (data not shown). In addition to this observation, IR-cSLO images collected from B6-albino mice appeared slightly different to the experienced examiner than those obtained from BALB/c. In the central imaging view, RIF were not as easily discerned in B6-albino as they were in BALB/c until they appeared peripherally. These observations suggest that some slight differences in RIF pathology development exist between these two albino strains, perhaps due to some microstructural anatomical differences in the retina, RPE or choroid, and iris tissues. Since these two strains are not genetically similar, perhaps there exists some differences in the incomplete conversion of melanin in the BALB/c vs. B6-albino strains.

Our group has observed over several years now that albino mice require substantially (~5x) less pupil dilation drop volume than pigmented mice prior to imaging (56). Such an observation makes one ponder that melanin pigment must be a good chelator of foreign substances including oxidized biochemical byproducts or cellular toxins. These anecdotal differences are intriguing and likely oversimplifying but perhaps there are more sensitive assays for delineating differences in pigmentation density, composition, or chelating ability within the two albino strains other than by visual appearance with the unaided eye. Developments in quantitative IRAF-cSLO (57), hyperspectral (58), directional and/or polarization sensitive SD-OCT (59–61) and photoacoustic tomography (62) may be useful for determining whether minuscule differences in RPE, choroidal, iris and uveal pigmentation exist between BALB/c and B6-albino strains that could improve our understanding of their profound differences in light susceptibility. If *in vivo* imaging cannot resolve the differences, then perhaps there are other means to accomplish this *via* electron microscopy or biochemical analysis. Melanin pigment density has been shown to decrease with age in humans and is believed to be a risk factor for age-related macular degeneration (63, 64) so it seems logical to step back and reconsider that albino susceptibility could be due in part to differences in incomplete melanin bio-composition and its ability to sequester free radicals and protect against toxic byproducts in the visual transduction cycle.

Recent advances in cSLO and SD-OCT instrument design have improved imaging resolutions and acquisition speeds such that transient events can be captured in real-time in a complimentary manner with a combined modality instrumentation. These instruments provide new insights into structure-function relationships that occur in response to a photopic light stimulus (38, 39, 65–67). Now referred to as intrinsic optophysiology or optoretinography, new studies conducted with these custom-integrated systems are capable of revealing time-resolved responses to brief light stimuli (68) or kinetic changes associated between light- and dark-adapted conditions (39, 67, 69, 70). In 2017, Zhang et al. demonstrated osmotic swelling of the outer retina in B6-albino mice in response to photobleaching from a brief two-minute exposure to blue light stimuli. The authors recorded prominent, and reversible, light scattering changes in the photoreceptor layer in response to this event (38). More recently, Kim et al., (67) demonstrated visualization of transient changes in the mouse photoreceptor layer due to the abrupt transition from light to dark conditions (67). These studies have actively recorded changes in OS reflectivity, either hypo- or hyper-, depending on whether light- or dark-induced stimuli were delivered to the retina of pigmented or albino mice.

Given these examples of functional imaging of the outer retina, we felt comfortable extracting additional data using the LRP analysis dissect the outer retina morphological dynamics from SD-OCT images for mice housed under BLE vs. DLE conditions. LRP analysis techniques have been previously reported and are useful for analyzing changes in back-reflected signal intensity is relation to retina axial depth; foremost correlating dark and light reflective band features to anatomical lamina within the retina (14, 40, 71). More recently, Zhang et al. used LRPs to display results from OCT optophysiology studies used to identify back reflective signal changes caused by osmotic tension due to phototransduction within the visual cycle (38). Others have used LRP analysis to elucidate the differences between OCT images of albino and pigmented mice, primarily for differentiating the apical and basolateral aspects of the RPE relative to the photoreceptor outer segment apical tips and Bruch’s membrane, respectively (11, 38, 60, 66, 72). Visualization of a very thin hyporeflective band presumably in the vicinity of the sub-retinal space is induced by diurnal light/dark changes and presumably related to mitochondrial respiration, altering of pH and movement of water *via* the RPE (67, 69).

These previous works enable us to confidently identify and correlate the outer retina anatomy associated with the bright and dark reflective bands observed in a typical grayscale OCT b-scan images that was carefully detailed in Figure 3. During this and in previous studies, we have observed reflective signal changes occurring at the proximal aspect of the photoreceptor outer segments in albino mice. More importantly, we have found that the altered morphology is not limited to mice as we have observed this feature in albino vs. pigmented rats as well (see Supplemental Figure S7). As opposed to the analog cSLO changes involving increasing RIF and AFF count, the proximal photoreceptor outer segment changes observed in OCT images appears as more of a digital event, either visibly present or absent. A quest to document this change in these three mouse strains was important for underscoring its validity as a predictor of light stress and possibly photoreceptor toxicity and degeneration. This development appears to be a steady-state event if animals remain exposed daily to elevated levels of illumination. In Figure 6, BLE is observed altering the OSS∠ after only 1 week in B6-albino vs. B6 mice. This alteration of signal reflectivity is reconfirmed the following week after 7 additional days of BLE. Upon return to dim lighting the following week for 7 days of DLE, the augmented OSS∠ is reversed in B6-albino. In comparison, pigmented B6 mice did not exhibit any substantial changes to the OSS∠ in response to the bright light stimulus challenge further validating this analysis approach for detecting light stress between albino and pigmented mice. These changes are like those reported by Zhang et al. where increased backscatter was observed originating from the base and tips of the OS following a 2-minute photobleaching causes 10% isomerization of rhodopsin in BALB/c retina (38). Rather than visualizing rapid, acute changes, our study differs by monitoring what occurs after many weeks of chronic, long-term photobleaching light exposure and continual rhodopsin isomerization of BALB/c, B6-albino and B6 mice retina. Perceivably, there is some dysfunction in the visual system of these albino mice that results in the build of isomerized rhodopsin and retinal esters, leading to a toxic buildup of byproducts that are altering the light scatter properties of the proximal photoceptor outer segments.

As shown in Figures 3 and 6, a change in signal reflectivity between the proximal photoreceptor outer segments and the IS-OS junction and distal photoreceptor outer segment tips amounts to a change in contrast between these three laminar entities. In a normal mouse not under light stress, OCT back-reflected signals present a light-dark-light-dark-bright signal banding that correspond to the OLM, IS, IS-OS, proximal and then distal photoreceptor outer segment regions as shown in Figure 3. As the photoreceptors and/or RPE become stressed, the banding is altered within the region of the photoreceptor outer segments and transitions from light-dark-light to light-less dark-light or light-light-light in the case of mild or severe stress, respectively, for example. Since demonstrating that this change is reversible after removing the BLE stimulus in B6-albino and not present in B6, we can define this hallmark feature as an indicator or light stress and depending on the currently unknown mechanism of induction, a potential indicator of visual cycle disfunction and/or homeostasis imbalance between the RPE and photoreceptors. Our previous work determined that similar changes in the outer retina are associated with stress in a mouse model with documented increased stress, the DJ-1 knockout mouse (7, 8).

While analyzing the OSS∠ data for Figure 6, it became apparent that a better method may exist for quantifying the changes in the proximal OS region. As opposed to using the signal slope, we recognized that one could simply use a relative calculation between neighboring lamina to accentuate the differences in contrast between the proximal OS and the IS-OS. We found that subtracting the proximal OS signal from the IS-OS signal amplitude was far less complicated and easier to perform than the OSS∠ analysis. Examples of the improvement in BALB/c mice can be seen in Figures 5A vs. 5B, where 100% (14/14) vs. 64% (9/14) of imaging time points were significant at detecting the reflective signal change for ΔOS vs. OSS∠ analysis, respectively. This observation is further supported in B6-albino mice (Figures 5D vs 5E), where 90% (9/10) and 40% (4/10) of imaging time points show that ΔOS outperformed OSS∠.

For various reasons vision researchers may occasionally wish to transfer a particular mutant line from one background strain to another. For example, one may wish to change from a BALB/c to B6-albino background to suppress or remove the light susceptible trait while still maintaining the albino characteristics. Alternatively, one may wish to transfer the mutant from a pigmented B6 background over to an albino to enhance the visualization characteristics of the model to be able to detect sub-apical fluorescent features in the RPE like Green Fluorescent Protein (GFP) expressing proteins or other sub-cellular constituents. It is anticipated that such a transfer would improve sensitivity by removing the strong attenuation factor from melanin pigment on the apical side of the mouse RPE cell. The work outlined here demonstrates why one must proceed with caution when transferring mice onto different backgrounds as this study demonstrates that B6-albino and B6 behave differently under the presence of low, human perceived, illuminance conditions. From the data presented here it is readily apparent that B6 and B6-albino are not equivalent strains and furthermore, nor are BALB/c and B6-albino, for the very same reasons. The B6-albino strain originally emerged from B6 mice as a spontaneous mutation and are recognized by JAX as a non-inbred, congenic strain to B6. Given the previous research and literature on this topic, one would expect B6-albino to behave in a similar manner as B6 but in fact, we found that B6-albino mice respond more similarly to BALB/c than they do B6, including the development of RIF, AFF, altered photoreceptor outer segment changes and photoreceptor degeneration.

Many questions come to mind when presented with this data. Has any animal model truly been adequately characterized if its retinal dynamics have not been documented over time in conjunction to environmental light exposure history? What could be the reason for the multi- or biphasic retinal degeneration response in the B6-albino strain? Have these mice reached their photon flux absorption threshold as hypothesized by Wenzel et al. (28)? What would have happened if the study would have continued? Presumably: 1) BALB/c and B6-albino would continue to degenerate, 2) B6 mice under BLE would continue to accumulate more AFF than their DLE counterparts, and 3) at some point, B6 mice would begin to exhibit an increasing rate of photoreceptor degeneration when approaching 3 years of age. We have imaged only a handful of naïve C57BL/6J mice (n~2; data not shown) at that age out of thousands imaged and both had substantial retinal pathology. Rounding out these questions would still take a significant investment in resources and time to complete but provide additional evidence to support these findings. Regardless, the current study has contributed some new insight into the retinal behavior of these three strains under two contrasting vivarium lighting conditions. The study has filled in some gaps pertaining to the retinal dynamics of these two albino mouse strains. Noteworthy is that it seems this data may provide new rationale for revisiting the cause and effect of light damage due to the differences observed between B6-albino, BALB/c and B6 strains of mice. It has also underscored the importance of knowing and documenting animal model genetic background and light history before, during and after experimental manipulations, and the need to perform semi-comprehensive “model characterization” assessments using non-invasive ocular imaging so that retinal dynamics are clearly established prior to commencement of pivotal experimental studies or the development of genetic models on any particular mouse strain whether albino or pigmented. Finally, the novel diagnostic, visual biomarker involving the proximal photoreceptor outer segments and quantitative analyses for said change has been put forward for recognizing when an albino mouse model in under light stress and is at risk for photoreceptor degeneration. Our results here have demonstrated that cSLO and OCT imaging used in conjunction with dynamic analysis of biomarkers can be used to further elucidate the differences between these three mouse strains and assist in recognizing when the pigmented epithelium and photoreceptors may be undergoing a homeostasis imbalance that can result in long-term stress to one or both entities.

## Supplementary Material

Supplemental Figure S1 **SUPPLEMENTARY FIGURE 1** Illustration of Analysis#4 measurements outer segment signal slope (OSS∠) from LRP SD-OCT B-scans.

Supplemental Figure S3 **SUPPLEMENTARY FIGURE 3** Illustration of Analysis#6 measurements for extracting the inner segment signal amplitude (ΔIS).

Supplemental Figure S2 **SUPPLEMENTARY FIGURE 2** Illustration of Analysis#5 measurements for extracting the outer segment signal amplitude (ΔOS).

Supplemental Figure S5 **SUPPLEMENTARY FIGURE 5** Scatter plot of the OS signal slope vs DLE-BLE-DLE challenge for B6-albino and B6 mice. Each dot represents the average slope from the 4 regional quadrants. Group averages shown as Mean+/−95% CI.

Supplemental Figure S4 **SUPPLEMENTARY FIGURE 4** Illustration of Analysis#7 measurements for extracting and parsing out individual lamina from the photoreceptor layer (PL) and adjacent retinal pigmented epithelium-Bruch’s membrane complex (RPEBM).

Supplemental Figure S6 **SUPPLEMENTARY FIGURE 6** Spearman’s Correlation and Predictive Power Score (PPS) Matrices for BALB/c, B6-albino, and B6 mouse strains.

Supplemental Figure S7 **SUPPLEMENTARY FIGURE 7** BLE-induced SD-OCT reflectivity changes in the proximal photoreceptor outer segment of an albino vs. pigmented rat.

## Figures and Tables

**TABLE 1:** Experimental study details including: the overall, Generalized Description between the two light treatment parameters (“DLE” & “BLE” for Dim and Bright Light Exposures, respectively), the type of mouse vivarium room where the mice were housed (“LI” for Low Illuminance & “SI” for Standard Illuminance), the cage rack location where the mice resided (“R”, “B” and “T” for Random, Bottom, or Top locations, respectively), the mean intracage cyclic light intensity parameters experienced by those mice residing at the reported cage rack locations (e.g. R, B or T) and vivarium (e.g. LI or SI), the mouse strains studied and post-baseline imaging timepoints in weeks following baseline at ~9 weeks of age.

Generalized Description	Vivarium Room Type (Abbrev.)	Cage Rack Location (Abbrev.)	Mean ±SD (lx) Intracage Illuminance (Abbrev.)	Mouse Strains (Grp.)	Post-baseline Imaging Timepoints (wks.)
DLE	Low Illuminance (LI)	Random (R)	1.2 ±1.7 lx (1lx)	B6 B6-albino BALB/c (A)	1,2,4, 7, 20, 44, 54, 57, 66, 68 1, 2.9, 4.3, 4.7, 7.4, 8.9, 10, 14, 18, 27, 29, 44, 49, 53, 58, 70 1, 2, 3, 4, 5, 6, 8, 10, 15, 18, 26, 40
	Standard Illuminance (SI)	Bottom (B)	7 ± 7 lx (7 lx)	BALB/c (B)	1, 2, 3, 4, 6, 8, 10, 12, 15, 20, 25, 35, 36, 48
BLE		Top (T)	151 ± 79 lx (150 lx)	B6 B6-albino BALB/c (C)	1, 2, 4, 20, 44, 54, 57, 66, 68 1, 2.4, 2.9, 4.3, 4.7, 7.4, 8.9, 18, 34, 44, 58, 70 1, 2, 3, 4, 6, 8, 10, 12, 15, 20, 25, 35, 36, 48

**TABLE 2:** Data analyses performed on cSLO and SD-OCT images (Units: #, total number counted within a 55°cSLO FOV; μm, thickness in micrometers; au, arbitrary units; gsu, grayscale units).

Analysis Number & Description	Data Source	Quantification	Measurement	Units	Abbrev.
1. Retinal infoldings	IR-cSLO	Population	Total count/55° FOV	#	RIF
2. Autofluorescent foci	BAF-cSLO	Population	Total count/55° FOV	#	AFF
3. Photoreceptor degeneration	SD-OCT B-scans	Morphology	ONL thickness	μm	ONL
4. Outer segment signal slope	Longitudinal Reflectance Profiles (LRPs) from SD-OCT B-scans	Slope of the back-reflected signal	Δy/Δx = (y_max_ − y_min_)/(x_max_ − x_min_)	au	OSS∠
5. Outer segment signal amplitude		Relative differences between back-reflected signals	Δ=IS/OS_max_ − OS_min_	gsu	ΔOS
6. Inner segment signal amplitude			Δ=IS/OS_max_− IS_min_		ΔIS
7. Photoreceptor Layer		Morphology	RPEBM-OLM	μm	PL+RPE
7a. Inner segments			IS/OS - OLM		IS
7b. Outer segments + RPE			RPEBM - IS/OS		OS+RPE
7b.1. Outer segments			OS – IS/OS		OS
7b.2. RPE			RPEBM – OS		RPE

## Data Availability

The raw data supporting the conclusions of this article will be made available by the authors, without undue reservation.
