# Supplementary figures and images for: A comparison of optophysiological biomarkers of photoreceptor stress and phototoxicity in BALB/cJ, B6 (Cg)-Tyrc-2J/J, and C57Bl/6J mouse strains
Source: Front Ophthalmol (Lausanne). Author manuscript; Available in PMC 2024 Apr 30. (PMC11057998; doi:10.3389/fopht.2023.1128311)

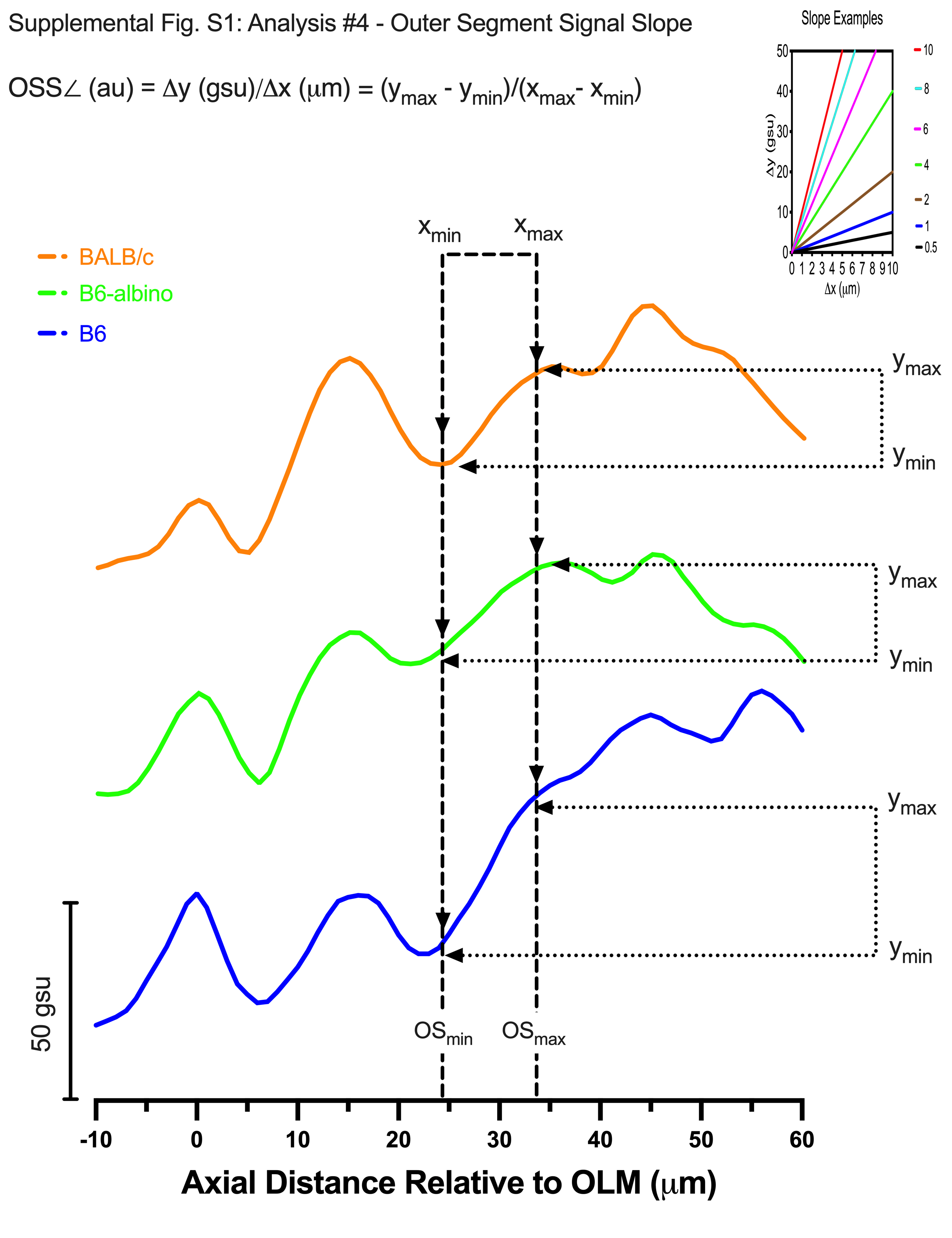

Supplement: Supplemental Figure S1 — SUPPLEMENTARY FIGURE 1 Illustration of Analysis#4 measurements outer segment signal slope (OSS∠) from LRP SD-OCT B-scans. [file NIHMS1987482-supplement-Supplemental_Figure_S1.tiff]

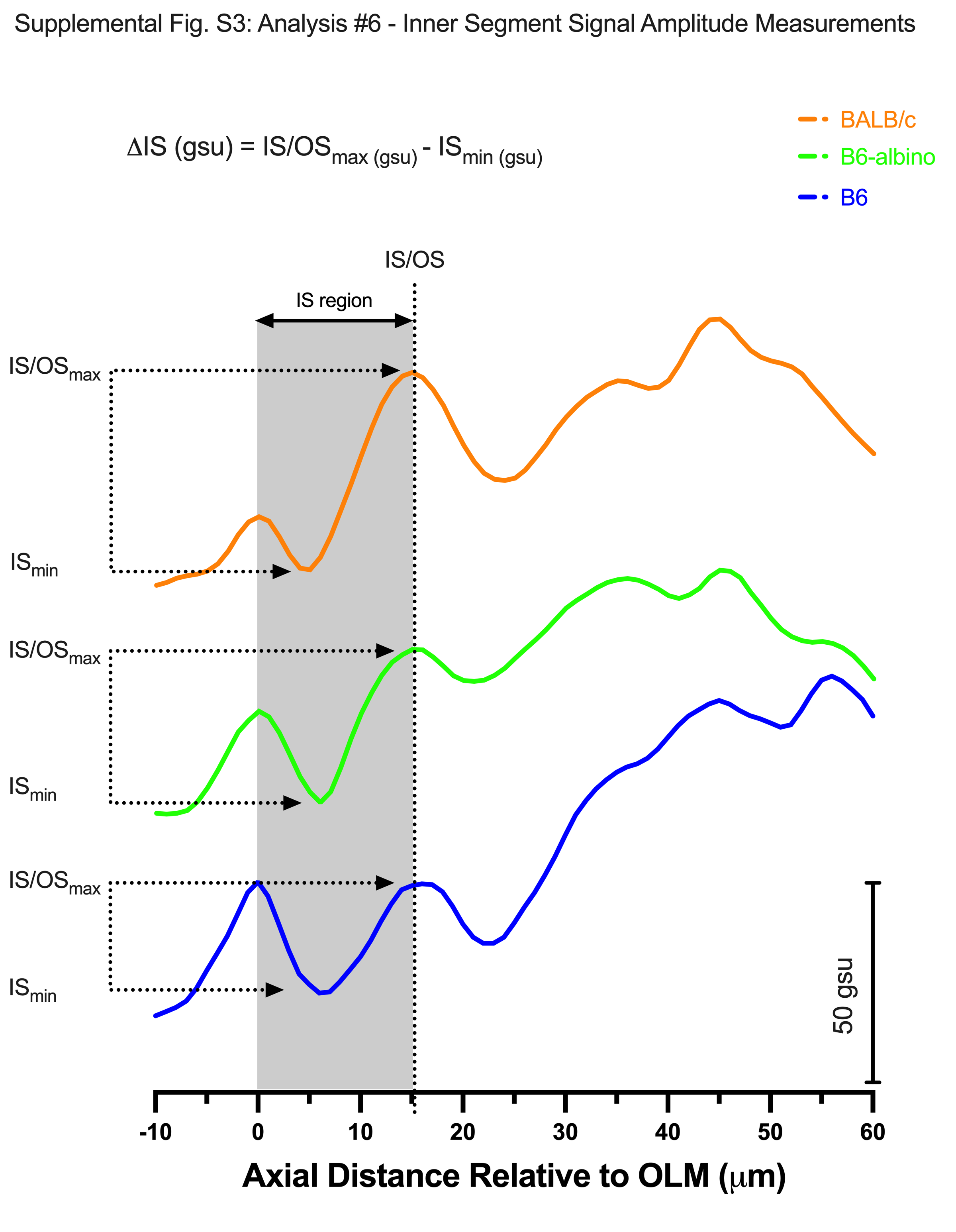

Supplement: Supplemental Figure S3 — SUPPLEMENTARY FIGURE 3 Illustration of Analysis#6 measurements for extracting the inner segment signal amplitude (ΔIS). [file NIHMS1987482-supplement-Supplemental_Figure_S3.tiff]

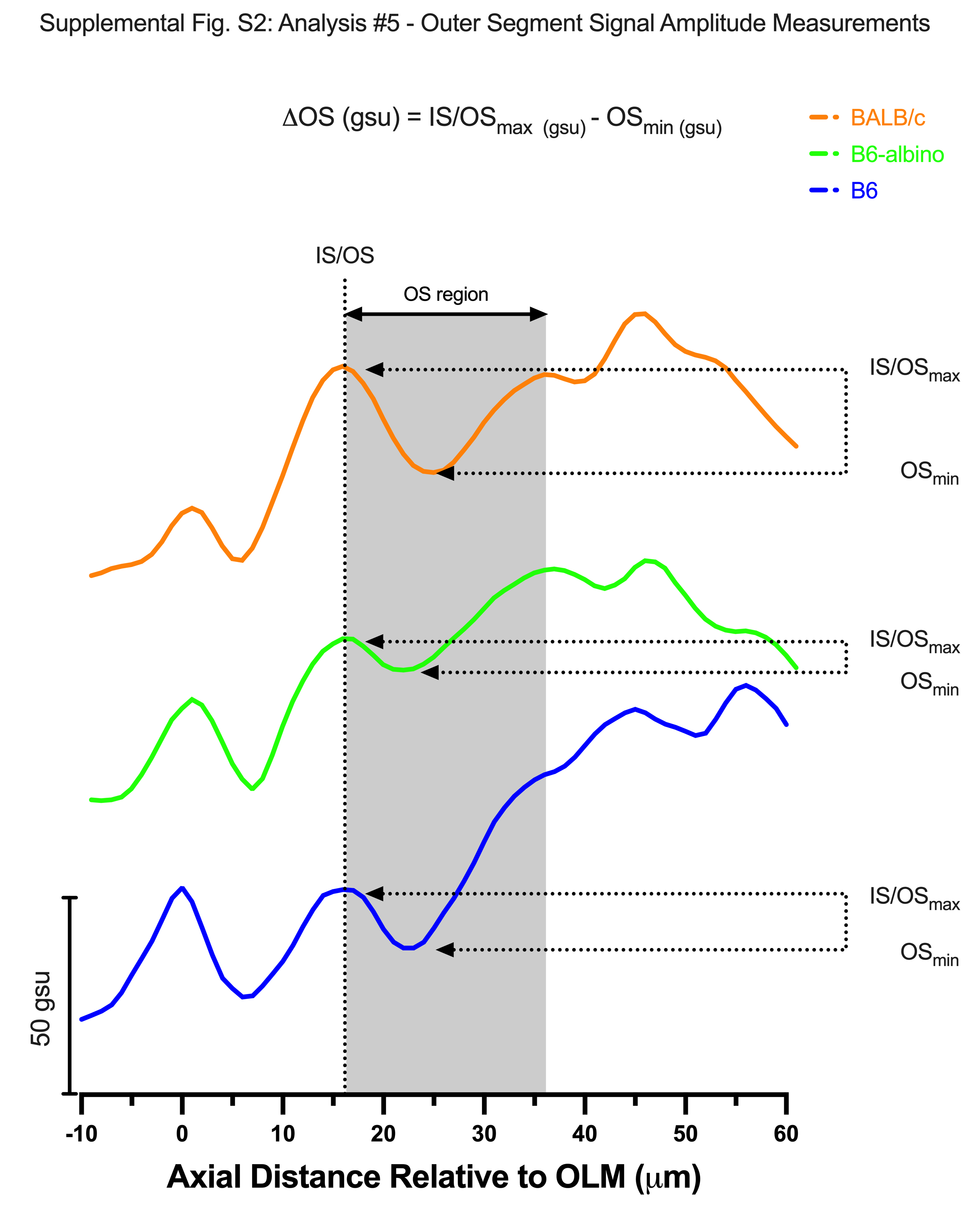

Supplement: Supplemental Figure S2 — SUPPLEMENTARY FIGURE 2 Illustration of Analysis#5 measurements for extracting the outer segment signal amplitude (ΔOS). [file NIHMS1987482-supplement-Supplemental_Figure_S2.tiff]

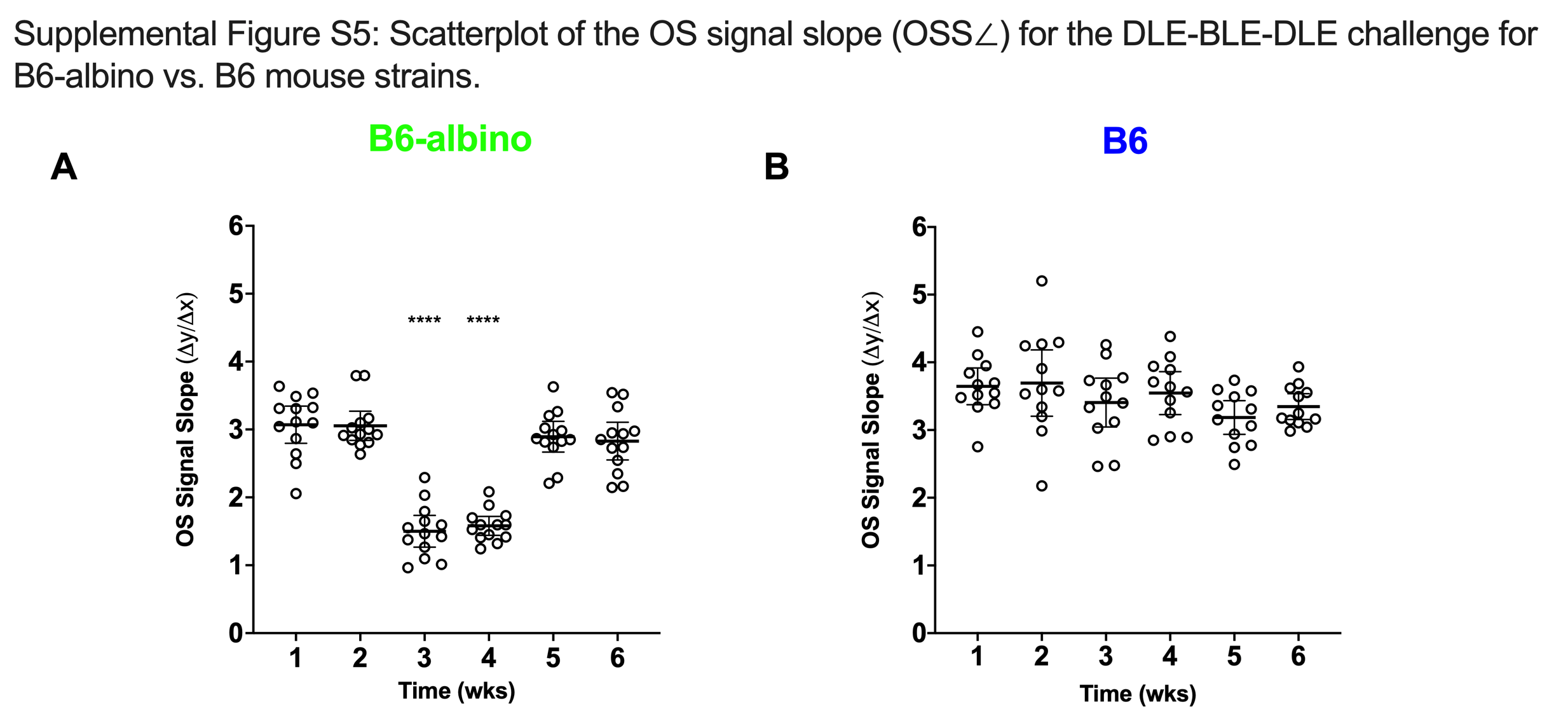

Supplement: Supplemental Figure S5 — SUPPLEMENTARY FIGURE 5 Scatter plot of the OS signal slope vs DLE-BLE-DLE challenge for B6-albino and B6 mice. Each dot represents the average slope from the 4 regional quadrants. Group averages shown as Mean+/−95% CI. [file NIHMS1987482-supplement-Supplemental_Figure_S5.tiff]

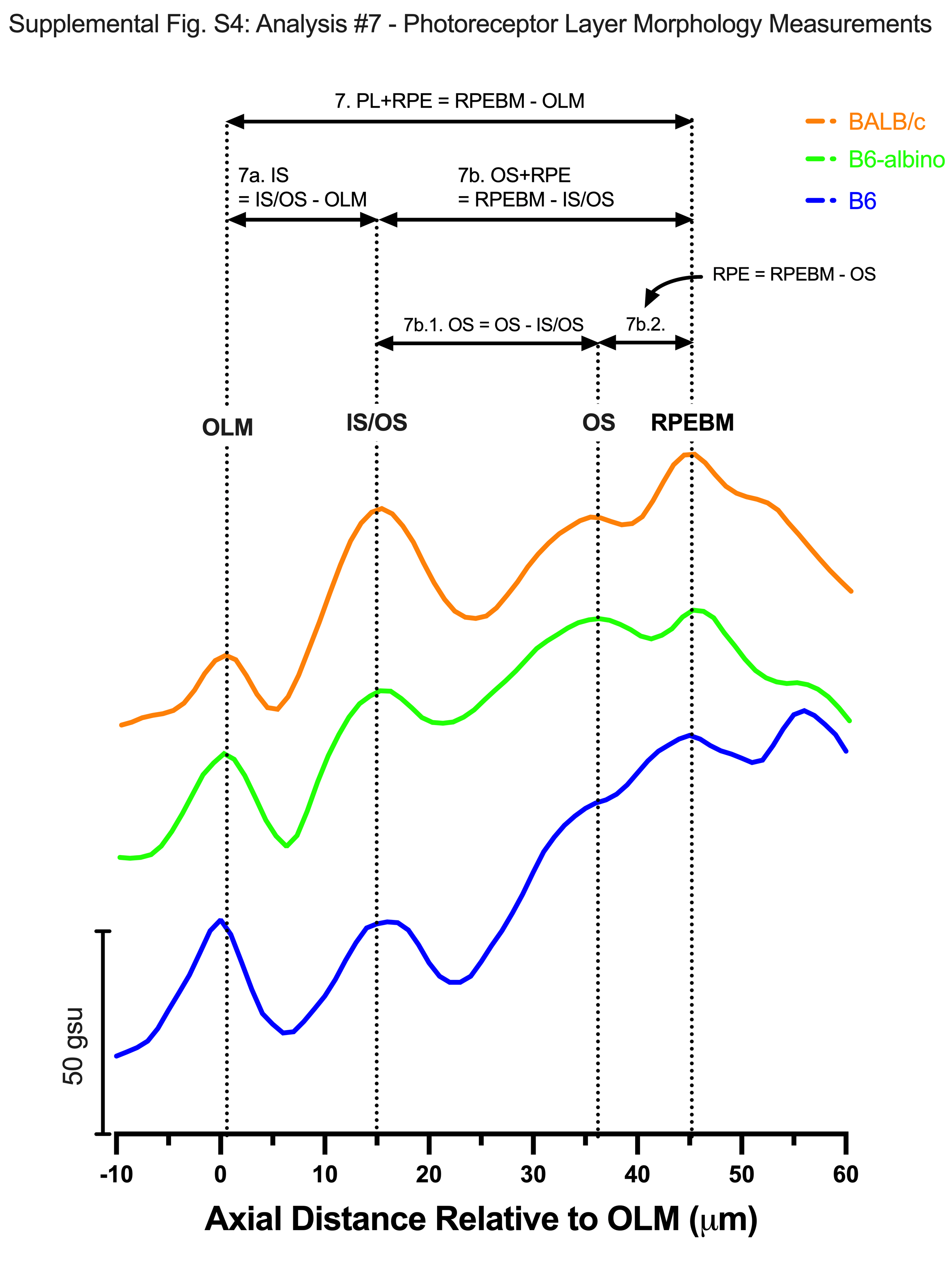

Supplement: Supplemental Figure S4 — SUPPLEMENTARY FIGURE 4 Illustration of Analysis#7 measurements for extracting and parsing out individual lamina from the photoreceptor layer (PL) and adjacent retinal pigmented epithelium-Bruch’s membrane complex (RPEBM). [file NIHMS1987482-supplement-Supplemental_Figure_S4.tiff]

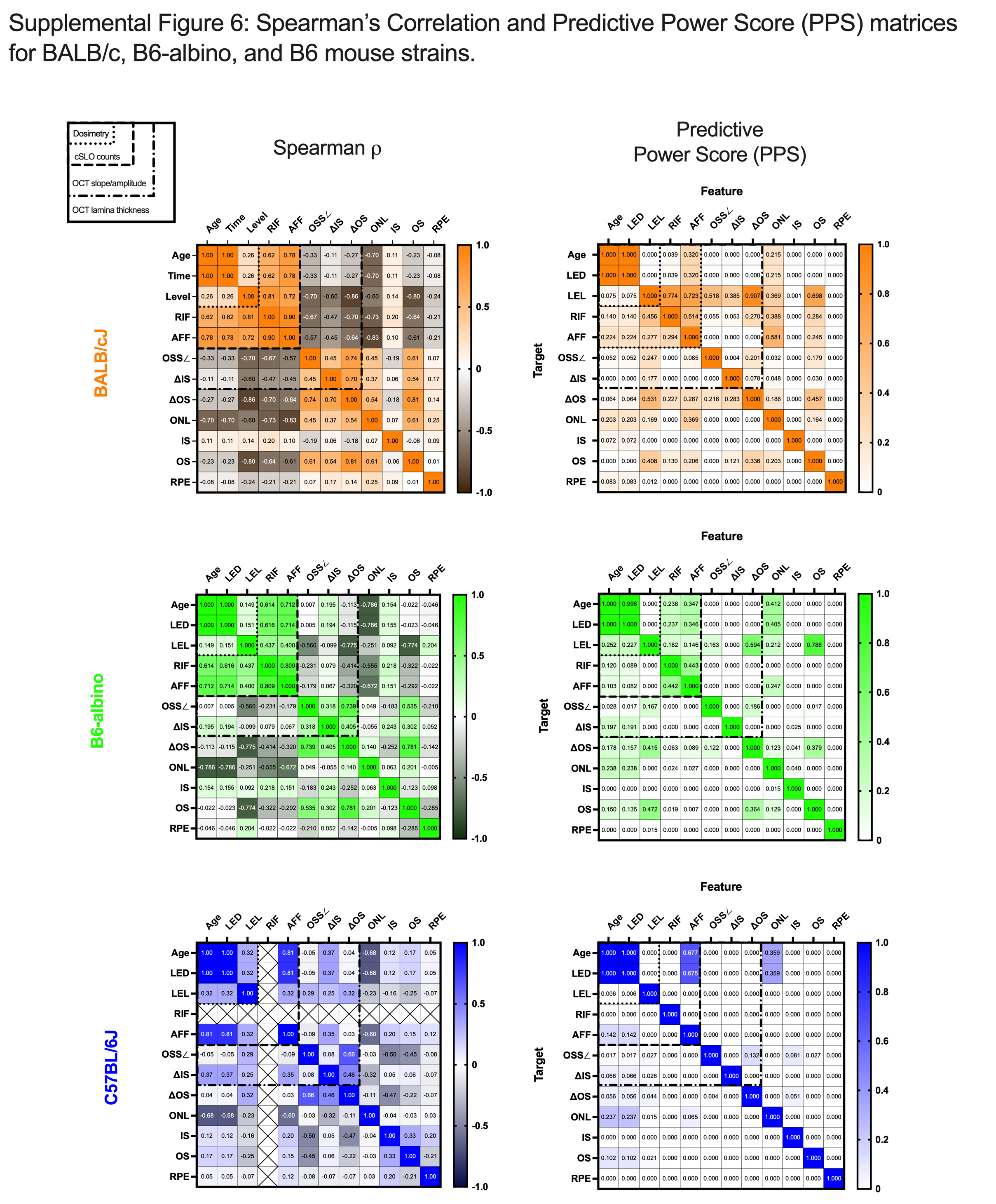

Supplement: Supplemental Figure S6 — SUPPLEMENTARY FIGURE 6 Spearman’s Correlation and Predictive Power Score (PPS) Matrices for BALB/c, B6-albino, and B6 mouse strains. [file NIHMS1987482-supplement-Supplemental_Figure_S6.tiff]

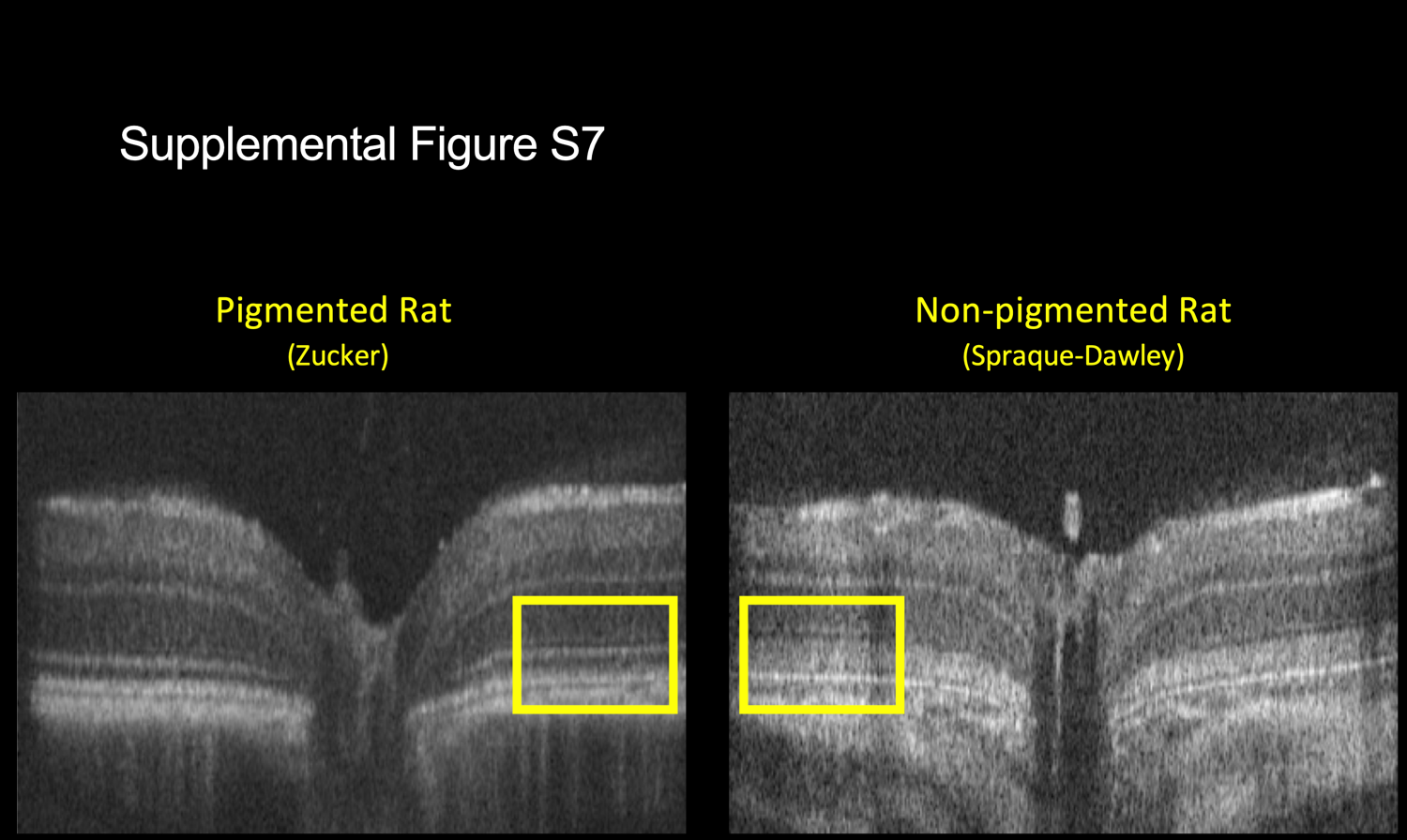

Supplement: Supplemental Figure S7 — SUPPLEMENTARY FIGURE 7 BLE-induced SD-OCT reflectivity changes in the proximal photoreceptor outer segment of an albino vs. pigmented rat. [file NIHMS1987482-supplement-Supplemental_Figure_S7.tiff]
